# Structure–function insights of natural *Ganoderma* polysaccharides: advances in biosynthesis and functional food applications

**DOI:** 10.1007/s13659-025-00496-w

**Published:** 2025-03-04

**Authors:** Zhou-Wei Wu, Xue-Fang Zhao, Chen-Xi Quan, Xiao-Cui Liu, Xin-Yu Tao, Yu-jie Li, Xing-Rong Peng, Ming-Hua Qiu

**Affiliations:** 1 State Key Laboratory of Phytochemistry and Natural Medicines, Kunming Institute of Botany, Chinese Academy of Sciences, Kunming, 650201 Yunnan People’s Republic of China; 2 University of Chinese Academy of Sciences, Beijing, 100049 People’s Republic of China

**Keywords:** *Ganoderma* polysaccharides, extraction techniques, structural characteristics, Bioactivity, biosynthetic pathways, Functional food applications

## Abstract

*Ganoderma* polysaccharides (GPs), derived from various species of the *Ganoderma* genus, exhibit diverse bioactivities, including immune modulation, anti-tumor effects, and gut microbiota regulation. These properties position GPs as dual-purpose agents for medicinal and functional food development. This review comprehensively explores the structural complexity of six key GPs and their specific mechanisms of action, such as TLR signaling in immune modulation, apoptosis pathways in anti-tumor activity, and their prebiotic effects on gut microbiota. Additionally, the structure–activity relationships (SARs) of GPs are highlighted to elucidate their biological efficacy. Advances in green extraction techniques, including ultrasonic-assisted and enzymatic methods, are discussed for their roles in enhancing yield and aligning with sustainable production principles. Furthermore, the review addresses biotechnological innovations in polysaccharide biosynthesis, improving production efficiency and making large-scale production feasible. These insights, combined with ongoing research into their bioactivity, provide a solid foundation for developing health-promoting functional food products that incorporate GPs. Furthermore, future research directions are suggested to optimize biosynthesis pathways and fully harness the health benefits of these polysaccharides.

**Graphical abstract:**

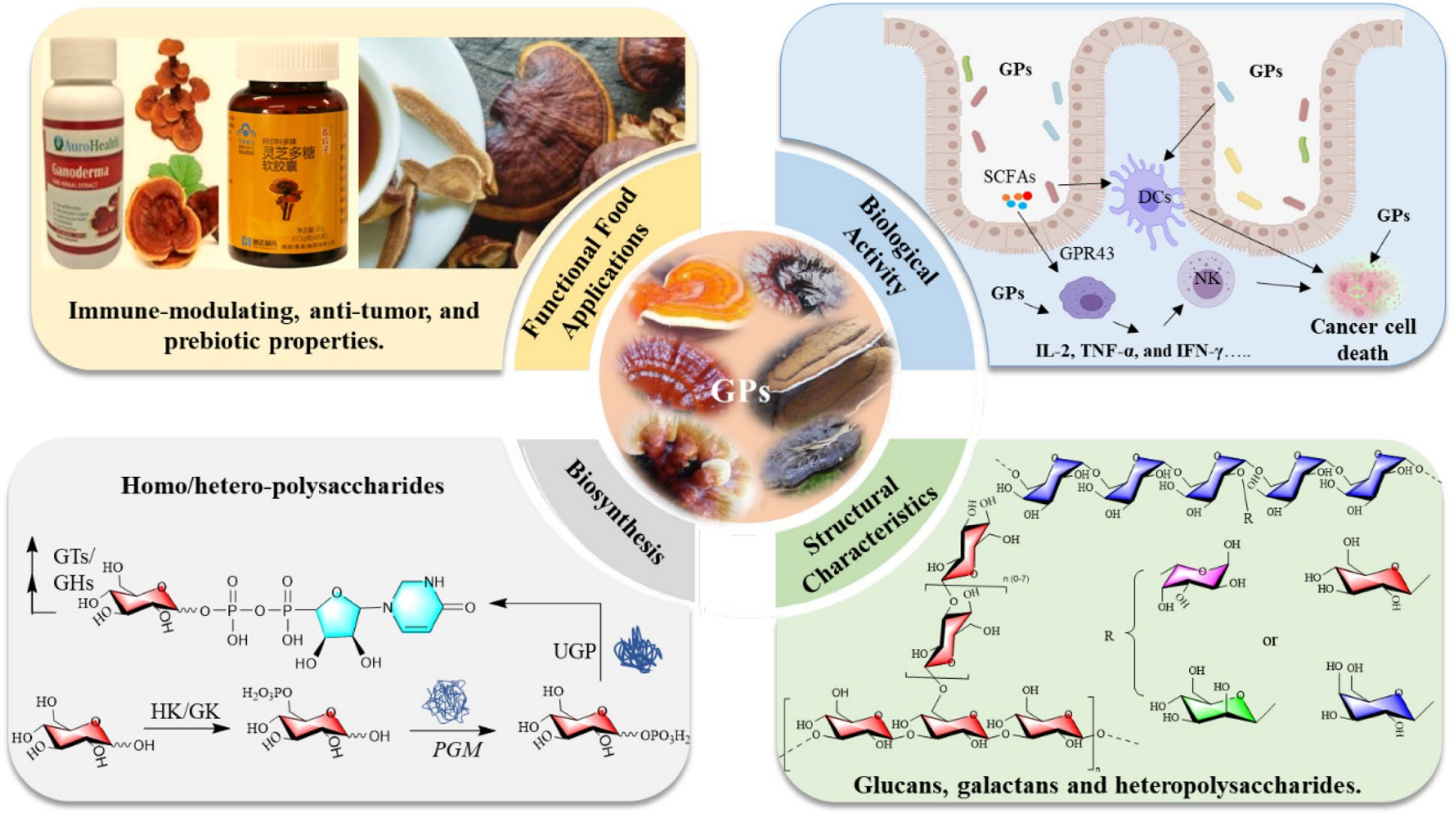

## Introduction

*Ganoderma* spp., a medicinal fungus belonging to the Basidiomycota division and Polyporaceae family, has been extensively studied due to its remarkable pharmacological properties. In traditional Chinese medicine (TCM), *Ganoderma* has long been regarded as a valuable medicinal herb, with records in ancient texts such as *Shen Nong Ben Cao Jing* (*The Divine Farmer's Materia Medica*), where it is noted for its ability to alleviate fatigue, treat respiratory ailments, and improve gastrointestinal health. In recent years, as the global influence of TCM has expanded, particularly in Europe and North America, the therapeutic value of *Ganoderma* has gained increasing recognition beyond Asia [1]. Modern pharmacological research has revealed that *Ganoderma* contains a wide range of bioactive compounds, including polysaccharides, triterpenes, sterols, peptides, and fatty acids [2]. These compounds exhibit a broad spectrum of biological activities, further reinforcing the global prominence of *Ganoderma* in pharmacopeias [3]. Among these, GPs have garnered significant attention due to their complex structures and diverse bioactivities, including immune modulation, anti-tumor effects, regulation of gut microbiota, and antioxidant properties [4]. GPs are now considered critical components in modern medicine and nutrition. According to recent market research, the global *Ganoderma* extract market is projected to reach USD 6.86 billion by 2024, with an estimated increase to USD 10.58 billion by 2029, representing a compound annual growth rate (CAGR) of 9.04% (Mordor Intelligence, 2024). This significant market growth reflects the rising demand for natural health-promoting products and highlights the immense potential of GPs for applications in functional foods, positioning them as promising candidates for dietary supplements, functional beverages, and health snacks [5].

The *Ganoderma* genus consists of over 200 species worldwide, with *G. lucidum*, *G. sinense*, *G. tsugae*, *G. applanatum*, *G. atrum*, and *G. leucocontextum* being the most studied for their polysaccharide [6]. Polysaccharides from these species are extracted using a variety of techniques, including hot water extraction, enzymatic extraction, ultrasonic-assisted extraction, and supercritical fluid extraction, resulting in diverse polysaccharide structures [7]. These polysaccharides exhibit complex structural characteristics, including high molecular weights ranging from several thousand to several million daltons, diverse monosaccharide compositions, various glycosidic linkages, and intricate spatial conformations [8, 9]. The complexity of these structures primarily arises from the biosynthetic pathways of GPs, where glycosyltransferases and glycosyl hydrolases polymerize nucleotide-sugar precursors to form the polysaccharide backbone, which is then modified by the addition of different monosaccharide units and branching structures through the action of other related enzymes [10]. The diversity in structure directly contributes to the wide range of biological activities exhibited by GPs, including immunomodulatory, anti-tumor, gut microbiota regulation, antioxidant, anti-inflammatory, and hepatoprotective effects [11–16]. Of all the species, *G. lucidum* has garnered the most attention, with reviews systematically summarizing its polysaccharide structures and biological functions, making it the most comprehensively studied species in the genus [17–19]. While reviews have made significant contributions, they tend to focus on specific aspects, such as polysaccharide structures or broad biological activity listings, lacking a comprehensive integration of structural characteristics, structure–activity relationships, biosynthetic pathways, and their potential applications in medicinal and functional food development.

A comprehensive investigation into the chemical structures, biological activities, biosynthetic pathways, and potential applications of polysaccharides derived from six *Ganoderma* species is essential for driving progress in this field. This review explores their mechanisms of interaction in immunomodulation, gut health regulation, and anti-tumor activity, alongside their structure–activity relationships. Moreover, the review systematically addresses recent biotechnological advancements, particularly the breakthroughs in overexpression of key biosynthetic genes, which have significantly enhanced the production capacity of *Ganoderma* polysaccharides, making large-scale application in both medicinal and functional food development.

## Extraction and structural characteristics of GPs

### Extraction of GPs

#### Content variation of GPs

*G. lucidum* is rich in various chemical constituents, including 0.7–1.8% ash, 21.83–27.78% crude fiber, 3.0–5.8% fat, 13.3–23.6% protein, 42.8–82.3% carbohydrates [20]. The contents of GPs undergo substantial dynamic variations depending on the growth stage and the specific part of the organism, including the mycelium, fruiting body, and spores. Mid-infrared (Mid-IR) and near-infrared (NIR) spectroscopy were used to assess polysaccharide content in *Ganoderma* mycelium from various sources, with contents ranging from 0.6% to 11.3% [21]. Four different *Ganoderma* strains grown on various substrates were investigated, and the polysaccharide content was found to range from 18.45 to 112.82 mg/g of dry weight [22]. Subsequently, The total polysaccharide content of *G. lucidum* at six different growth stages was systematically assessed using the total carbohydrate assay kit [23]. The results indicated that the total polysaccharide content gradually increased from stages S1 to S3 (2.5–3.2%), suggesting that this stage is critical for polysaccharide accumulation. The highest polysaccharide content in *G. tsugae* was observed during the early developmental stage, particularly at primordium formation (S1, 1.5%), with the content gradually decreasing as the growth stages progressed [24]. These findings suggest that the early growth stages of *Ganoderma*, particularly stages S1 to S3, are crucial for polysaccharide accumulation. Moreover, *Ganoderma* samples from different regions in China showed that regional differences significantly influence the structure and content of their structural types. Specifically, β-1,3-glucan and β-1,3,6-glucan were identified as the primary structural components, with polysaccharide content ranging from 5.5 to 18.5 mg/g dry weight [25]. Recent studies employing hyperspectral imaging (HSI) technology in conjunction with machine learning models have facilitated the non-destructive detection of polysaccharide content in *Ganoderma* fruiting bodies [26]. These technologies not only improve the accuracy of polysaccharide content prediction but also provide real-time monitoring tools for determining the optimal harvest time for *Ganoderma*.

#### Hot water and alkaline extraction

Polysaccharides serve as a crucial bioactive component within *Ganoderma*, thereby underscoring the necessity for their efficient extraction for subsequent functional applications. The detailed extraction procedures are presented in Table 1. Hot water extraction (HWE) is one of the most common methods for extracting GPs. HWE for polysaccharides involves using high temperatures to disrupt the cell walls through thermal expansion and contraction [27]. This process allows the polysaccharides to be released from the cell matrix and dissolved into the surrounding hot water. The extraction process of GPs, typically conducted at 50 °C–100 °C for 1.5–5 h with 2–3 repetitions, is a relatively simple method conducive to large-scale production [28]. However, this process is characterized by its low efficiency, with yields ranging from 0.21% to 2.36% [25, 29]. While optimizing extraction conditions (e.g. elevated temperature, extended duration) can augment yield, it simultaneously results in increased energy consumption. Additionally, temperatures above 150 ℃ for more than 15 min induce a conformational change in the β-glucan structure that may result in reduced biological activity [7, 30]. Alkaline extraction (AE) uses chemical reagents, including NaOH, KOH, to break down cell walls and increase the yield of polysaccharides [31]. This procedure is commonly conducted after HWE. Alkali-soluble polysaccharides from *Ganoderma* were extracted using 6–8% NaOH, resulting in a significant yield increase ranging from 2.1% to 8.2% [32, 33]. Scanning electron microscopy (SEM) observations revealed that alkaline treatment disrupts the fibrous structure of *Ganoderma* cell walls, facilitating the release of polysaccharides [34, 35].

**Table 1:** Extraction methods and parameters of polysaccharides extraction from *Ganoderma* spp

Resources	origin	Extraction methods	Extraction conditions	Process highlights	Activity	Yield (%)	References
*G. lucidum*	fruiting bodies	HWE	Liquid–solid ratio 1:15 with 100 μL α-amylase and 100 μL cellulose, stirred at 130 rpm for 2 h at 90 °C water bath	Simple and cost-effective; suitable for large-scale production	–	0.21–1.85%	[25]
	spore powder				–	0.19–1.21%	
	fruiting bodies	AE	Residues re-extracted with 1 M NaOH solution containing 20 mM NaBH_4_, stirred for 1 h at 25 °C	Improves yield	–	0.67–1.05%	
	spore powder				–	0.55–1.02%	
*G. lucidum*	mycelial	HWE	Liquid–solid ratio 1:20 for 2 h at 100 °C water bath	Superior health safety profiles and reduced environmental risks	–	2.59%	[29]
*G. lucidum*	Dried fruiting bodies (Fujian, China)	HWE	Time: 137 min, Temperature: 66 °C, Liquid–solid ratio 35 mL/g	Optimized via RSM, fractionated into GLP40, GLP60, GLP80	GLP80 shows the highest antioxidant activities (DPPH, reducing power)	2.44%	[47]
*G. lucidum*	Fruiting bodies	HWE	Time: 230 min, Temp: 95 °C, Extraction cycles: 5	Optimized extraction conditions via RSM for enhanced yield and bioactivity	Significant immunomodulatory and antioxidant activities	1.45%	[48]
*G. lucidum*	Mycelium and Fruiting bodies	AE	Temp: 100 °C, Time: 3 h, 6% NaOH c, Liquid–solid ratio: 20 ml/g	High yield under optimal conditions	–	4.96%	[32]
*G. tsugae*	Mycelium	AE	2% NaOH at 25 °C, Precipitated by acetone	Exhibits semiflexible chain behavior with significant excluded volume effects	–	2.1%	[33]
*G. lucidum*	Fruiting bodies	AE	60.1 °C, 77.3 min, 5.1% NaOH, 21.4 mL/g	Significant improvement in yield compared to hot water extraction; breaks down fiber structure	Enhanced immune function, particularly in NK cell activity	8.21	[34]
*G. lucidum*	fruiting bodies	UAE	Ultrasonic power: 590–600 W, Time: 58–60 min, Temperature: 40–81 °C	Significantly higher yield and reduced energy consumption, enhanced antioxidant activity	DPPH scavenging rate: 55.44% at 75 µg/ml, FRAP: 0.0183 mmol Fe^2+^/l	0.52–0.81%	[36, 37]
*G. lucidum*	Fruiting bodies	UAE	Ultrasonic power: 320 W, Temp: 70 °C, Time: 34 min	Enhanced extraction efficiency with reduced time and energy consumption, maintaining high antioxidant activity	High antioxidant activity (DPPH and ORAC)	2.78%	[38]
*G. lucidum*	fruiting bodies	UAE	Ultrasonic power: 210 W, Extraction temperature: 80 °C, Liquid/solid ratio: 50 mL/g, Extraction time: 100 min	Better antioxidant activity compared to HWE	Higher antioxidant activity (DPPH and reducing power)	0.63%	[39]
*G. lucidum*	fruiting bodies	UAEE	Enzyme concentration: 3%, pH: 5.5, Temperature: 45 °C, Time: 30 min, Ultrasonic power: 480 W	Combined use of Viscozyme and Chitinase, enhanced extraction efficiency	IC50 > 512 μg/mL for cancer cell lines	3.21%	[40]
*G. lucidum*	fruiting bodies (logs-cultivated, Japan)	SWE	Temperature: 373–463 K, Pressure: 10.0 MPa, Extraction Time: 30–60 min, Liquid -Solid Ratio: 10:1 to 20:1	Maintains β-glucan structure, β-glucan content: 40%-45%	–	–	[49]
*G. lucidum*	fruiting bodies	HCWE	Temperature: 373–463 K, Pressure: 4.0 MPa	Optimized to maintain bioactive polysaccharide structure	–	–	[50]
*G. lucidum*	fruiting bodies	CPTE	Temperature: 100 °C, Flow rate: 28 L/h, Particle size: 2 mm	Significantly higher yield compared to HWE and UAE methods; Efficiently extracts high-molecular-weight polysaccharides	High immunomodulatory activity (NO, TNF-α, IL-6)	2.04%	[44]
*G. lucidum*	Fruiting bodies	DESs	Solid–liquid ratio: 1:31 g/mL, Temp: 78 °C, Time: 69 min	DESs provide enhanced solubility and selective extraction of polysaccharides	High antioxidant activity (DPPH assay)	9.472%	[46]
*G. lucidum*	Fruiting bodies	UCE	Ultrasonic power: 671 W, Temp: 48 °C, Time: 45 min, Intermittent-running ratio: 5.5/L, Solid–liquid ratio: 1:12.5	Enhanced extraction efficiency and antioxidant activity	High antioxidant activity (DPPH scavenging rate 53.63%)	47.87 mg/ml (concentration)	[41]
*G. lucidum*	Spores	MCAE	Solid–liquid ratio: 5 g/g, Milling time: 20 min, Solution/material ratio: 20 mL/g, Extraction time: 130 min	Optimized extraction with higher yield and lower temperature compared to HRE	Significant antioxidant activity (DPPH scavenging)	5.92%	[43]

Hot water extraction (HWE), Alkaline Extraction (AE), Ultrasonic-assisted extraction (UAE), Ultrasonic-Circulating Extraction (UCE), Ultrasound-Assisted Enzymatic Extraction (UAEE), Subcritical water extraction (SWE), continuous phase transition extraction (CPTE), Hot Compressed Water Extraction (HCWE), Ternary Deep Eutectic Solvent (DESs), Mechanochemical-Assisted Extraction (MCAE)

#### Ultrasonic extraction

Ultrasonic extraction, a contemporary technique characterized by its efficiency, speed, low-temperature requirements, and solvent conservation, is frequently employed for the extraction of GPs. The ultrasonic-assisted extraction (UAE) method, optimized with extraction parameters of 40–81 °C and 590–600 W, effectively balances yield and antioxidant activity, producing GPs yields ranging from 0.52% to 2.7% [36–39]. Furthermore, In vitro antioxidant activity assays revealed that the polysaccharides obtained through UAE demonstrate notable DPPH radical scavenging activity, comparable to that of the traditional antioxidant vitamin C, and surpass those extracted using HWE. Moreover, UAE is often used in conjunction with other biochemical reagents to enhance yields. For instance, Ultrasonic-assisted enzymatic extraction (UAEE) was used to extract polysaccharides from Vietnamese red reishi mushrooms. [40]. The optimal conditions were identified as pH 5.5, 45 °C for 30 min, and 480 W, employing Viscozyme and Chitinase enzymes, which resulted in a yield of 3.2%. An ultrasonic circulation extraction technique combined with ultrafine grinding (UCE) was developed, yielding a concentration of *Ganoderma lucidum* polysaccharides (GLPs) at 47.87 mg/mL [41]. In tests assessing antioxidant activity, this technique demonstrated a notable DPPH radical scavenging rate of 53.63%, suggesting its considerable potential for antioxidant applications. Additionally, an ultrasonic/microwave-assisted extraction (UMAE) method was developed for GLPs. Under optimal conditions, UMAE markedly enhanced the extraction efficiency of these polysaccharides, yielding a 115.6% increase over traditional HWE and a 27.7% rise compared to UAE [42].

#### Other extraction methods

With the advancement of extraction technologies, a growing number of new methods have been applied to the extraction of GPs, leading to increased efficiency and yield [27]. A mechanochemical-assisted extraction (MCAE) technique was developed for efficiently extracting polysaccharides from *Ganoderma* spores [43]. This method, in comparison to the hot reflux extraction approach, not only markedly enhanced the extraction yield to 5.92% at a reduced temperature but also diminished the extraction duration by approximately 45.8%. Continuous phase transition extraction (CPTE) technology significantly outperforms traditional methods, achieving a polysaccharide extraction rate that is 3.34 times higher than HWE and 2.68 times higher than UAE [44]. Furthermore, the GPs extracted through CPTE demonstrate a higher molecular weight and significant immunoregulatory activity. Microwave-assisted extraction (MAE) has also emerged as a highly efficient method for extracting GPs, producing notable results in shorter timeframes. Studies show that MAE can enhance the extraction yield of GPs to approximately 7.7%, with extraction times reduced to nearly 90% of those required by conventional HWE [45]. The ternary deep eutectic solvents (DESs) system, composed of choline chloride, guaiacol, and lactic acid in a specific molar ratio, was optimized under carefully controlled conditions, leading to an impressive extraction efficiency for GPs, with the yield reaching as high as 9.5% [46].

The extraction of GPs is essential for their functional applications. Contemporary techniques, including UAE, Subcritical water extraction (SWE), and CPTE, markedly enhance both the yield and purity of the extracted polysaccharides compared to conventional HWE. These techniques also reduce extraction duration and minimize structural damage to maintain bioactivity, underscoring their significant potential for broader applications.

### Structural characteristics of GPs

Polysaccharides have been extensively studied in various parts of *Ganoderma* species, including fruiting bodies, mycelium, spores, and fermentation broth [51]. These polysaccharides are primarily crude extracts, often containing heterogeneous mixtures of polysaccharides, glycoproteins, and other macromolecules [52, 53]. Among these, only a subset has been purified and structurally characterized as homogeneous polysaccharides with defined chemical structures.

**Fig. 1:**
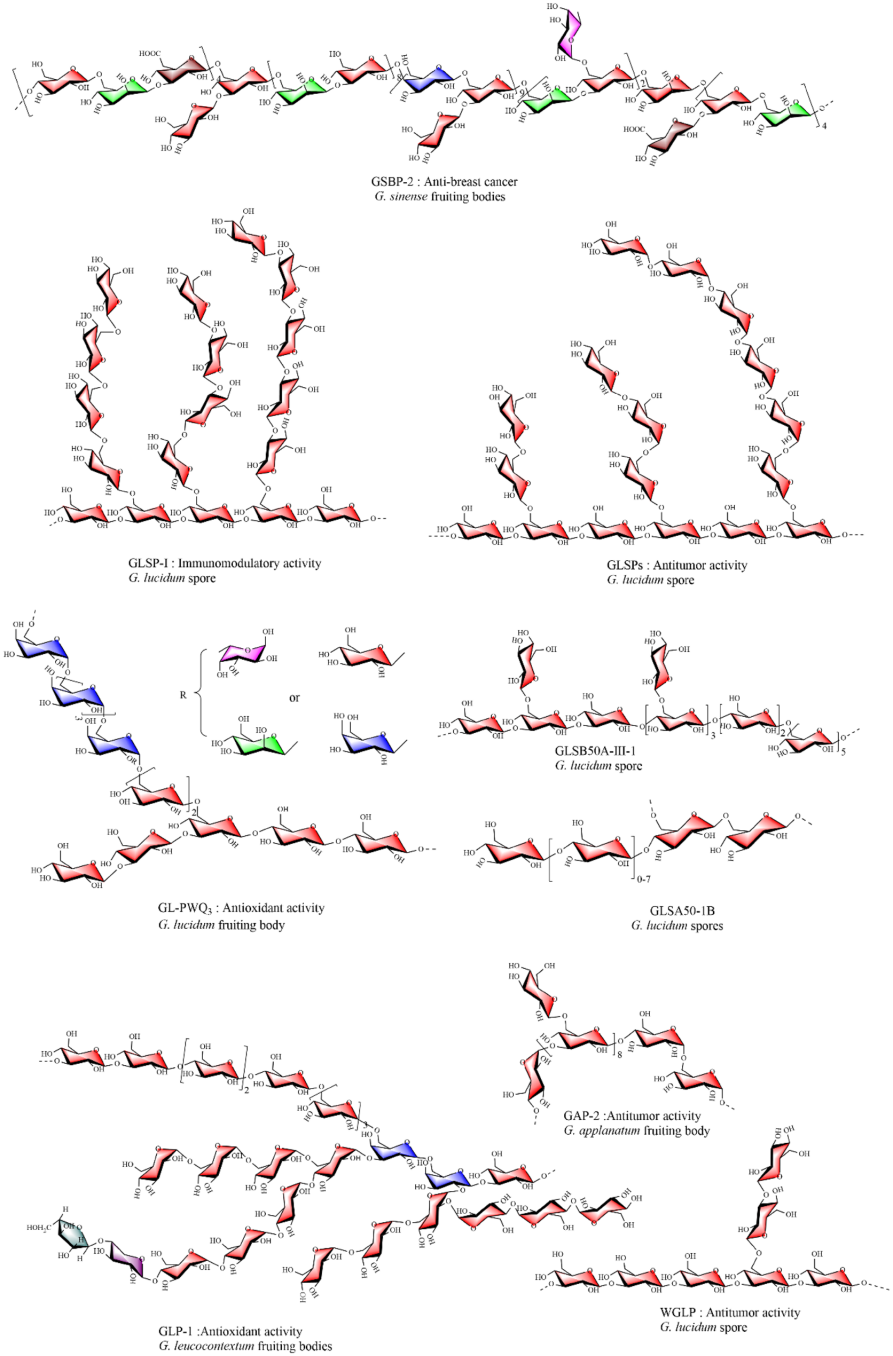

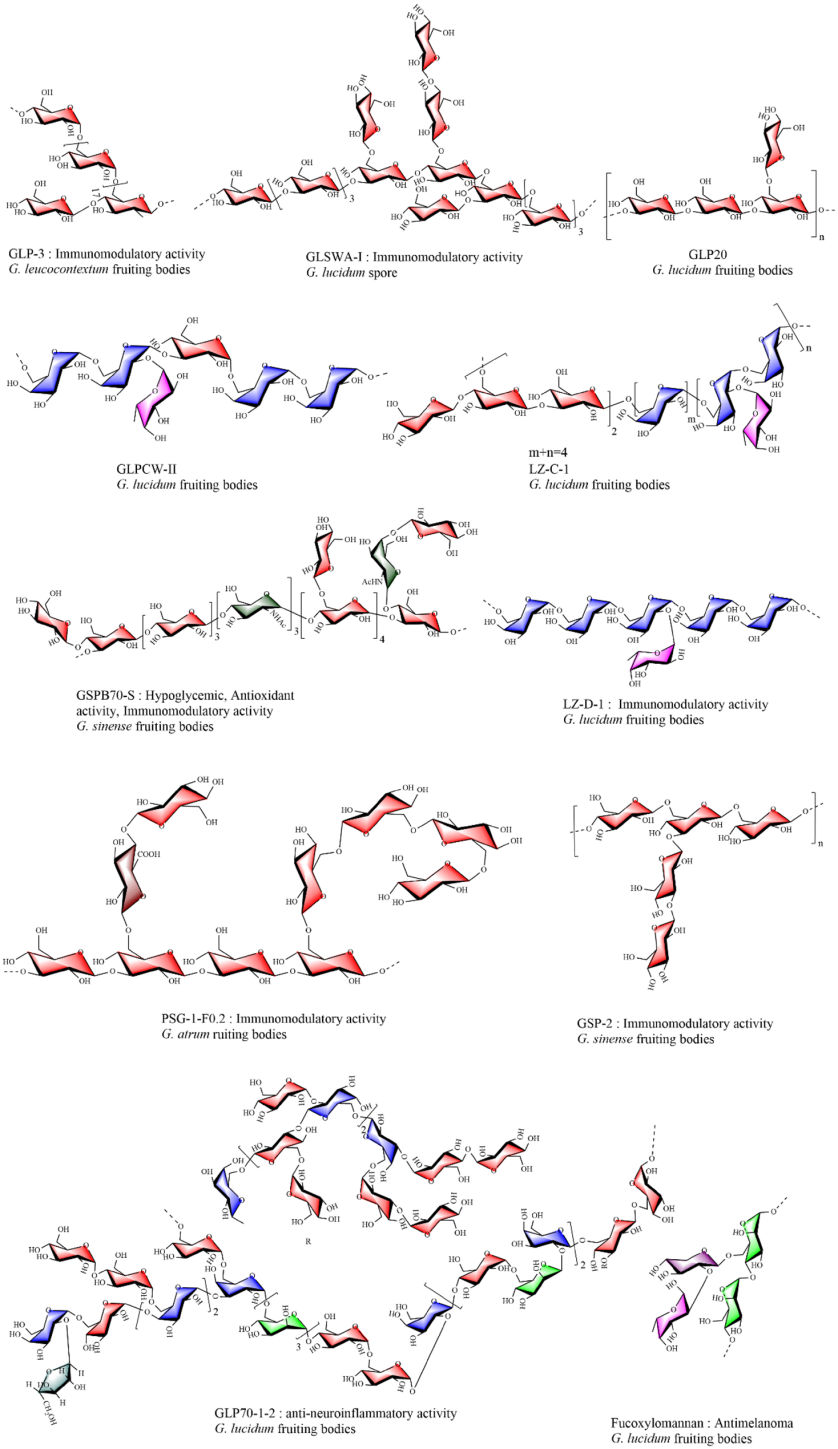
The proposed chemical structures of the homopolysaccharides and heteropolysaccharides of *Ganoderma* spp

As summarized in Table 2 and Fig. 1, homogeneous polysaccharides derived from *Ganoderma* encompass diverse structural types, such as β-D-glucans, α-D-glucans, α-D-galactans, and heteropolysaccharides [54]. Despite their structural diversity, many *Ganoderma* polysaccharides share conserved features, including β-(1 → 3)-D-glucan backbones with β-(1 → 6)-linked branching, which are commonly observed across different species [55]. In addition, conserved galactan backbones composed of α-(1 → 6)-linked galactose residues are also present in certain polysaccharides. These conserved motifs play critical roles in their immunomodulatory and antitumor activities [56]. However, structural diversity arises from variations in monosaccharide composition, glycosidic linkages, molecular weights, branching patterns, and stereochemical configurations. For example, polysaccharides often include both α- and β-glycosidic linkages (e.g., α-D-Glcp and β-D-Glcp), which contribute to their conformational flexibility and biological activities [57]. These structural features, both conserved motifs and diverse modifications, collectively contribute to their broad spectrum of biological activities.

**Table 2:** Structural characterization from *Ganoderma* spp

Resources	Extraction methods	Name	Structure feature	Monosaccharide composition	Molecular weight (kDa)	References
*G. lucidum* fruiting bodies	AE with 10% KOH, and fractionation by ion exchange chromatography	GLC-1	Linear (1 → 3)-β-D-Glc backbone	Glc:Man at molar (%) ratio of 95.3:3.5		[75]
		GLC-2	Mixture of (1 → 3)-α-D-Glcp, (1 → 3)-β-D-Glcp, and (1 → 4)-α-D-Manp	Glc:Man: Xyl:Fuc at molar (%) ratio of 69.4:17.4:7.9:5.3		
		GLC-3	Glucans with a main chain of β-(1 → 4)-D-Glcp, with branches linked at the O-3 or O-6	Glc:Man:Gal:Xyl at molar (%) ratio of 95.5:1.1:3.4	13	
*G. lucidum* fruiting body	HWE, membrane ultrafiltration, and gel column chromatography (TSK G4000 PW)	GL-PWQ3 (glycopeptide)	Glucogalactan with a backbone of (1 → 6)-α-D-Galp, (1 → 6)-β-D-Glcp, (1 → 4)-β-D-Glcp, with branches linked at the O-3 and O-2. Fucp-(1 → , Manp-(1 → , → 2)-Manp-(1 → , Glcp-(1 → , → 3)-Glcp-(1 → , → 6)-Glcp-(1 → , → 4)-Glcp-(1 → , → 3,6)-Glcp-(1 → , → 4,6)-Glcp-(1 → , Galp-(1 → , → 2)-Galp-(1 → , → 6)-Galp-(1 → , → 2,6)-Galp-(1 → , at molar ratio of 5.07:4.96:2.07:20.72:5.08:11.26:12.16:5.79:2.00:1.42:1.04:22.95:5.48	Glc:Man:Gal: Fuc:Rha at molar ratio of 107:50:16:11:1	2.4	[56]
*G. lucidum* fruiting body	HWE, ethanol precipitation, further purified by QFF anion-exchange column	GLP-1	Galactoglucan with a flexible random linear. α-D-Fucp-(1 → , β-D-Glcp-(1 → , → 3)-β-D-Glcp-(1 → , → 6)-β-D-Glcp-(1 → , → 6)-α-D-Galp-(1 → , → 3, 6)-β-D-Glcp-(1 → , → 4, 6)-β-D-Glcp-(1 → at molar ratio (%) of 5.5:10.3:13.1:38.2:20.6:5.2:7.1	Man:Glc:Gal at molar (%) ratio of 4.9:63.5:26.2:5.4	107	[59]
		GLP-2	β-D-glucan with a spherical conformation. α-D-Fucp-(1 → , β-D-Glcp-(1 → , → 3)-β-D-Glcp-(1 → , → 6)-β-D-Glcp-(1 → , → 6)-α-D-Galp-(1 → , → 3, 6)-β-D-Glcp-(1 → , → 4, 6)-β-D-Glcp-(1 → at molar ratio (%) of 3.6:13.4:13.7:50.3:6.7:6.9:5.4	Man:Glc:Gal at molar (%) ratio of 1.6:90.6:7.8	19.5	
*G. lucidum* fruiting bodies	HWE, ethanol precipitation	GLP20	β-D-glucan with a backbone of 1 → 3)-β-D-Glcp, a branch linked at a O-6, forming triple-helix conformation in water. Glcp (1 → , → 3)-Glcp (1 → , 3,6)-Glcp (1 → at molar ratio of 1.00:2.07:1.01	Glc	3750 (0.9% NaCl), 1350 (DMSO)	[62]
*G. lucidum* fruiting bodies	Air-dried; extracted with 95% EtOH; further purified using DEAE-Sepharose and Sephacryl S-300 columns	LZ-D-1	Fucogalactan with a backbone of (1 → 6)-β-D-Gal, with a branch linked at the O-2. → 6)-Galp-(1 → , Fucp-(1 → , → 2,6)-Galp-(1 → at molar ratio of 4.01:0.96: 1.00	Fuc:Gal:Glc at a molar ratio of 1:5:1	28	[69]
*G. lucidum* fruiting bodies	Air-dried, extracted with 95% EtOH, further purified using DEAE-Sepharose and Sephacryl S-300 columns	LZ-C-1	Galactoglucan with a backbone of (1 → 6)-β-D-Glcp, (1 → 3)-β-D-Glcp, (1 → 6)-α-D-Galp, with two branch linked at the O-4 and O-2. Fuc-(1 → , → 2,6)-Gal-(1 → , → 6)-Gal-(1 → , Glc-(1 → , → 4,6)-Glc-(1 → , → 3)-Glc-(1 → , → 6)-Man-(1 → at molar ratio of 0.93:1.00:3.89:1.91:2.03:2.23:0.32		7.0	[70]
*G. lucidum* fruiting bodies	HWE, ethanol precipitation, further purified by DEAE- Sepharose and Sephacryl S-300 HR	GLPCW-II (glycopeptide)	Galactoglucan with a backbone of (1 → 6)-α-D-Galp, (1 → 3)-β-D-Glcp, with a branch linked at the O-2 position. → 3)-Glcp-(1 → , Fucp-(1 → , → 6)-Galp-(1 → , → 2,6)-Galp-(1 → at a molar ratio of 1:1:3:1; with minor amounts of → 2)-Galp-(1 → , Fucp-(1 → , and → 6)-Manp-(1 →	Glc:Fuc:Gal at a molar ratio of 1.00:1.09:4.09	12	[71]
*G. lucidum* fruiting bodies	HWE, alkaline extract (10% KOH), ethanol precipitation	fucoxylomannan	Fucoxylomannan with a backbone of (1 → 6)-α-D-Manp, with α-L-Fucp-(1 → 2)-β-D-Xylp as a branch linked at the O-6. Fucp-(1 → , 2 →)-Xylp-(1 → , Manp-(1 → , 3 →)-Glcp-(1 → , 4 →)-Manp-(1 → , 6 →)-Glcp-(1 → , 4,6 →)-Manp-(1 → , 3,6 →)-Glcp-(1 → at area (%) ratio of 25:24:3:1:21:trace:25: trace	Fuc:Xyl:Man:Glc at molar (%) ratio of 24.0:25.0:48.3:2.7	35.9	[73]
*G. lucidum* fruiting bodies	HWE, ethanol precipitation, further purified by DEAE-52 and Sephacryl S-100	GLP70-1–2	Galactoglucomannan with complex structure. Fucp-(1 → , Glcp-(1 → , → 4)-Glcp-(1 → , → 6)-Glcp-(1 → , → 3,6)-Glcp-(1 → , → 6)-Manp-(1 → , → 2)-Galp-(1 → , → 6)-Galp-(1 → , → 4,6)-Galp-(1 → , → 2,6)-Galp-(1 → at a molar ratio of 1.3:8.0:6.9:3.8:2.5:4.8:3.8:1.0:3.3:2.1	Main contained Man, Gal, Glc with minor Fuc	6.2	[76]
*G. lucidum* fruiting bodies	Degreasing, HWE, AE (1 M KOH), water-insoluble precipitation (neutralized by acetic acid)	GLPs	β-D-glucan with a backbone of (1 → 3)-β-D-Glcp, with branches linked at the O-2 and O-6 position, forming flexible chain. Glcp (1 → , → 3)-Glcp (1 → , → 4)-Glcp (1 → , 3,6)-Glc (1 → at molar (%) ratio of 4.40:91.17:3.31	Glc	133	[77]
*G. lucidum* spore	acid extraction (0.1 M H_2_SO_4_), and alcohol precipitate	GLSPs	The β-D-glucan with a backbone of (1 → 3)-β-D-Glcp, with branches linked at the O-6. Glcp-(1 → , → 3)-Glcp-(1 → , → 4)-Glcp-(1 → , → 6)-Glcp-(1 → , → 3,6)-Glcp-(1 → at molar ratio of 12.66:13.31:13.53:14.15	Glc:Man:Gal:Fuc at molar (%) ratio of 87.5:0.7:3.1:8.7		[78]
*G. lucidum* spore	HWE, ethanol precipitation, remove starch, further purification by DEAE-52 and Sephadex G100	GLSP-I	β-D-glucan with a backbone of (1 → 3)-β-D-Glcp, with three branches linked at the O-6 and consisted of (1 → 3)-β-D-Glcp, (1 → 6)-β-D-Glcp, and β-D-Glcp-(1 →	Glc	128.0	[61]
*G. lucidum* spores	HWE, ethanol precipitation, further purification by DEAE-Sepharose, Sephacryl S-500 HR	GLSB50A-III-1	β-D-glucan with a backbone of 1 → 3)-, (1 → 4)-, and (1 → 6)-β-D-Glcp, with two branches linked at O-6, forming a globular sphere conformation in aqueous solution. Glcp-(1 → , → 3)-Glcp-(1 → , → 4)-Glcp-(1 → , → 6)-Glcp-(1 → , → 4,6)-Glcp-(1 → , 3,6)-Glcp-(1 → at molar ratio of 4.5: 1.7: 1.6: 5.3: 3.2: 1.0	Glc	193.0	[79]
*G. lucidum* spores	HWE, ethanol precipitation, DEAE-cellulose chromatography, Sephacryl S-300 HR column chromatography	WGLP	β-D-glucan with a backbone of (1 → 3)-β-D-Glcp, with a branch linked at a O-6. Glcp-(1 → : → 6)-Glcp-(1 → : → 3,6)-Glcp-(1: → 6)-Glcp-(1 → at molar ratio of 1.0:1.2:1.1:2.7	Glc	15	[80]
*G. lucidum* spores	HWE, precipitation, further purified by DEAE-Sepharos, Sephacryl S-300 HR	GLSWA-I	β-D-glucan with a backbone of 1 → 3)-, (1 → 4)-, and (1 → 6)-β-D-Glcp, with three branches linked at the O-4 and O-6. Glcp (1 → , → 3)-Glcp (1 → , → 4)-Glcp (1 → , → 6)-Glcp (1 → , 4,6)-Glcp (1 → , 3,6)-Glcp (1 → at molar ratio of 2.9:3.5:1.0:3.0:1.8:1.0	Glc	157	[63]
*G. lucidum* spores	HWE, ethanol precipitation, further purified by DEAE-52 and Sephacryl S-300 HR	GLSA50-1B	β-D-glucan with a backbone of (1 → 6)-β-D-Glcp, with 1 → 4-β-D-Glcp of 1–7 numbers as a branch linked at the O-4. Glcp (1 → , → 4)-Glcp (1 → :6)-Glcp (1 → , 4,6)-Glcp (1 → at molar (%) ratio of 21:22:38:19	Glc	103	[64]
Broth of *G. lucidum*	Ethanol precipitation, deproteinization, further purified by DEAE- Sepharose andSephadex G200	GLP-2	Heteropolysaccharide with a backbone of (1 → 6)-α-D-Galp, (1 → 3)-α-D-Glcp, with a branch linked at the O-2 position. → 4)-Galp-(1 → , → 4,6)-Galp-(1 → , Manp-(1 → , → 6)-Manp-(1 → , Glcp-(1 → , → 4)-Glcp-(1 → , Araf-(1 → , Rhap-(1 → at molar ratio of 56:15:4:8:2:6:7:2	Gal:Man:Glc:Ara:Rha at a molar ratio of 103:17:12:10:3:3	12	[81]
*G. sinense* fruiting bodies	AE, ethanol precipitation, deproteinization, Further purified using DEAE-52 and Sephacryl S-100 columns	GSBP-2	Heteropolysaccharide composed of α-L-Fucp-(1 → , β-D-Glcp-(1 → , β-D-GlcpA-(1 → , → 3)-β-D-Glcp-(1 → , → 3)-β-D-GlcpA-(1 → , → 4)-α-D-Galp-(1 → , → 6)-β-D-Manp-(1 → , and → 3,6)-β-D-Glcp-(1 → at a ratio of 1.0:6.3:1.7:5.5:1.5:4.3:8.0:7.9	Man:GlcA:Glc:Gal:Fuc at a molar ratio of 9.1:2.2:12.2:3.7:1.0	11.5	[82]
*G. sinense* fruiting bodies	AE (0.5 M NaOH), further purified using DEAE-Sepharose Fast Flow column	GSPB70-S	Heteropolysaccharide with a backbone of → 3)-β-D-Glcp-(1 → 4)-α-D-GlcpNAc-(1 → 4)-α-D-Manp-(1 → 3)-β-D-Glcp-(1 → , forming triple helix. Glcp-(1 → , → 4)-Galp-(1 → , → 3,4)-Glcp-(1 → , → 4,6)-Manp-(1 → , GlcpNAc-(1 → , → 4)-GlcpNAc-(1 → at molar ratio of 10.7:1.2:4.5:3.8:1.0:3.2	Glc:GlcNAc:Man at molar ratio of 12.90:3.70:2.26:1.00	2.87	[74]
*G. sinense* fruiting bodies	HWE, followed by ethanol precipitation, deproteinization, and Sephadex G-100 column chromatography	GSP-2	Protein-bound glucan with a backbone of (1 → 4)-β-D-Glcp and (1 → 6)-β-D-Glcp, with side chains of (1 → 3)- and terminal-linked β-D-Glcp at the O-3 position. Glcp-(1 → , → 3)-Glcp-(1 → , → 4)-Glcp-(1 → , → 6)-Glcp-(1 → , → 3, 6)-Glcp-(1 → molar (%) ratio: 27:16:15:22:10	Mainly composed of Glc with small amounts of Man and Gal		[83]
*G. applanatum* fruiting body	HWE, precipitate with ethanol, further purification DEAE-52, and Sephadex G-100	GAP-2	The glucan with a backbone of -4)-α-D-Glcp-(1 → [3,6)-β-D-Glcp-(1 →]_8_–4)-α-D-Glcp-(1 → -6)-α-D-Glcp-(1 → , with branches linked at the O-6, forming a spherical conformation in aqueous solutions. Glcp-(1 → , → 4)-Glcp-(1 → , → 6)-Glcp-(1 → , → 3,6)-Glcp-(1 → at molar ratio of 10.65:2.24:1.00:7.85	Glc:Man:Gal:GlcA:Xyl at a molar ratio of 82.0:5.6:4.3:4.2:3.9	21.3	[65]
*G. leucocontextum* fruiting bodies	HWE, ethanol precipitation, deproteinization, ultrafiltration membrane, further purified by DEAE-Sepharos, Sephacryl S-300 HR	GLP-3	β-D-glucan with a backbone of (1 → 4)-α-Glcp, (1 → 4,6)-β-D-Glc, with a β-Glcp-(1 → branch, forming triple-helix conformation in water. Glcp-(1 → , → 4)-Glcp-(1 → , → 4,6)-Glcp-(1 → at molar ratio of 1:17.5:1.5	Ara:Xyl:Man:Glc at molar ratio of 2.4:3.3:0.8:92.7:0.8	159.7	[57]
*G. leucocontextum* fruiting bodies	HWE, ethanol precipitation, deproteinization, further purified by ultrafiltration membrane, DEAE-Sepharose, Sephacryl S-300 HR	GLP-1	Heteropolysaccharide with a backbone of 1 → 3)-, (1 → 4)- β-D-Glcp, and (1 → 6)-β-D-Galp, with two complex branches at O-2 and O-6 in the Galp, no triple-helix. T-Araf-(1 → , Arap-(1 → , T-Fucp-(1 → , → 3,4)-Rhap-(1 → , → 4)-Xylp-(1 → , T-Glcp-(1 → , T-Manp-(1 → , → 3)-Glcp-(1 → , → 4)-Glcp-(1 → , → 6)-Galp-(1 → , → 4,6)-Galp-(1 → , → 4,6)-Glcp-(1 → , → 2,6)-Galp-(1 → at molar ratio of 3.28: 1.09: 0.88: 1.49: 3.25: 11.29: 1.46: 12.75: 25.89: 10.14: 11.97: 3.73: 8.69: 4.09	Man: Glc: Gal: Xyl: Ara at a molar (%) ratio of 7.02: 60.85: 12.00: 8.58: 7.51	6.3	[66]
		GLP-2	Heteropolysaccharide with β-linkages, including GlcUA and GalUA residues	Man:GlcUA:Glc:Gal: Xyl at a molar ratio of 17.95:3.24:50.75:6.08:12.79:9.19	14.1	
*G. atrum* fruiting bodies	HWE, ethanol precipitation, further purification using Superdex-G200 prep	PSG-1-F0.2	Heteropolysaccharide with a backbone of (1 → 3)-β-D-Glcp, with branches linked at the O-6, forming spherical conformation. Mainly composed of Glcp-(1 → , → 3)-Glcp-(1 → , → 2)-Manp-(1 → , → 4)-Glcp-(1 → , → 6)-Glcp-(1 → , → 6)-Galp-(1 → , → 3,6)-Glcp-(1 → , → 2,6)-Galp at area (%) ratio of 22.4:17.7:2.9:3.2:22.6:8.5:20.7:2.1	Glc:GlcA:Man at area (%) ratio of 73.8:15.3:5.7:5.2	12.73	[60]

#### Glucans and heteroglucans

Glucans are a major class of GPs, accounting for more than 40% of the total polysaccharide content [37]. The intricate structure of glucans is driven by several key factors, including molecular weight, glycosidic types, degree of branching (DB), and spatial conformation [9]. β-D-glucans are the most common polysaccharides found in *Ganoderma*, with molecular weight ranging from 1 × 103 Da to 5.5 × 10⁶ Da, primarily forming triple-helical and linear conformations, and the glycosidic types include β-(1 → 3), β-(1 → 4), and β-(1 → 6) linkages [58, 59]. The (1 → 3)-β-D-glucopyranosyl residues, which form the backbone of β-glucan, are frequently characterized. Typically, these main chains consist of 2–6 repeating units, with an average of 1–3 branches at the O-6 positions. The branching patterns include various forms of β-(1 →), β-(1 → 3), β-(1 → 4), and β-(1 → 6) glucans with 1–7 repeat units, as well as other glycosidic linkages such as β-(4 →)-D-GlcpA-(1 →) and terminal (T)-α-D-Glcp. These components collectively constitute highly branched β-D-glucans and β-D-heteroglucans [60, 61]. For instance, the GLP20 has a backbone composed of three (1 → 3)-β-D-Glcp units with branches linked at the O-6 position, forming a triple-helix conformation in water [62]. In addition to the 1,3-β-D-Glcp backbone, β-D-glucans are widely distributed in various combinations with (1 → 3), (1 → 4), and (1 → 6) linkages, exhibiting significant structural diversity and complexity. For example, the main chain of GLSWA-I consists of 1,3-β-D-Glcp, 1,4-β-D-Glcp, and 1,6-β-D-Glcp residues, with branches composed of β-D-Glcp-(1 → 4)-β-D-Glcp-(→ and β-D-Glcp-(→ residues attached at the O-6 and O-4 positions, resulting in a DB of 0.44 [63]. The backbone of GLSA50-1B is composed of 1,6-β-D-Glcp units, interspersed with 1–7 repeating 1,4-β-D-Glcp units as side chains linked at the O-4 position [64]. Additionally, glucans comprising mixed β-D- and α-D-glucan residues have also been identified including GAP-2 and GLP-3 [57, 65]. The backbone of GLP-3 is primarily composed of α-D-glucan and β-D-glucan residues in a molar ratio of 18:1, whereas GAP-2 contains these residues in a molar ratio of 3:8. Heteroglucans, primarily composed of Glc with minor sugars such as Ara, Gal, and Xyl, have been identified in *Ganoderma*. A notable example is GLP-1, which features a main chain of 1,3-β-D-Glcp and 1,4-β-D-Glcp with an inserted 1,6-α-D-Galp residue. This sequence is further complicated by the presence of two intricate side chains at the O-2 and O-4 positions of the Galp residues [66]. The structural diversity of glucans, encompassing mixed β-D- and α-D-glucan residues, heteroglucans, as well as variations in glycosidic linkages, space formation, and molar ratios, highlights the intricate architecture of GPs. These intricate structural features are key to defining their unique biological activities and potential applications.

#### Galactans and heterogalactans

Galactans and heterogalactans constitute another prominent structural class of polysaccharides found in *Ganoderma*. The primary structural characteristic of these polysaccharides is a backbone composed of 1,6-α-D-Galp units, with side chains including various non-reducing sugars, such as T-α-L-Fuc, T-α-D-Galp, T-α-D-Manp, and T-α-D-Galp-(1 → 6)-α-D-Galp-(1 → 4)-β-D-Glcp-(1 →), attached to the O-2 positions [56, 67, 68]. A homogeneous galactan from *Ganoderma* fruiting bodies, identified as LZ-D-1, has a molecular weight of 2.80 × 104 Da and a backbone composed of five 1,6-α-D-Galp repeat units with T-α-L-Fucp side chains attached at the O-2 position [69]. In vitro cell assays have demonstrated that LZ-D-1 stimulates the proliferation of mouse spleen lymphocytes, indicating its potential to enhance immune activity. LZ-C-1 was characterized by a main chain of 1,3-β-D-Glcp, 1,4,6-β-D-Glcp, and 1,6-α-D-Galp residues, with T-α-L-Fucp linkages at the O-2 position of the 1,6-α-D-Galp residues forming branches [70]. Additionally, the glycopeptides found in *Ganoderma* feature sugar chains containing heterogalactans, with main chains composed of 1,6-α-D-Galp, 1,4,6-β-D-Glcp, and 1,4-β-D-Glcp. Examples include GL-PWQ3 and GLPCW-II, which have molecular weights of 2.4 × 104 Da and 1.2 × 104 Da, respectively [56, 71]. The polysaccharide portion of GL-PWQ3 mainly consists of 1,6-α-D-Galp, 1,6-β-D-Glcp, and 1,4-β-D-Glcp residues, with side branches linked at the O-3 position with T-Glcp and 1,3-Glcp, and at the O-2 position with T-Fucp, T-Manp, or T-Glcp. The pronounced immunomodulatory and antioxidant properties of GL-PWQ3 highlight its therapeutic potential, further emphasizing the importance of *Ganoderma*-derived galactans and heterogalactans in health promotion and disease prevention.

#### Other types of polysaccharides

Galactoglucomannan was discovered with a molecular weight of 8.0 × 104 Da from the fruiting bodies of *G.* lucidum, which they named GLP70-1–2 [72]. GLP70-1–2 possesses a complex main chain structure, consisting of → 6)-α-D-Glcp-(1 → 6)-β-D-Galp-(1 → [6)-β-D-Manp-(1]_3_ → 4)-α-D-Glcp-(1 → 6)-α-D-Glcp-(1 → 2)-β-D-Galp-(1 → [4)-α-D-Glcp-(1 → 6)-β-D-Manp-(1 → 2)-β-D-Galp-(1]_2_ → 6)-β-D-Glcp-(1 → 6)-β-D-Glcp-(1 → , with two highly complex side chains attached at the O-4 and O-3 positions of the 1,6-β-D-Galp residues. Another homogeneous polysaccharide, a fucoxylomannan with a molecular weight of 3.5 × 104 Da, was identified from the fruiting bodies of *G. lucidum* using alkaline extraction methods [73]. The structure of fucoxylomannan is characterized by a main chain composed of 1,4-α-D-Manp repeating units. These units are flanked by side chains of T-α-D-Fucp-(1 → 2)-β-D-Xylp-(1 → , which are attached at the O-6 positions of the 1,4-α-D-Manp units. Additionally, from *G. sinense*, a polysaccharide named GSPB70-S was identified, with a main chain consisting of → 3)-β-D-Glcp-(1 → 4)-α-D-GlcpNAc-(1 → 4)-α-D-Manp-(1 → 3)-β-D-Glcp-(1 → [74]. GSPB70-S exhibits multiple biological activities, including antioxidant, immunomodulatory, and α-glucosidase inhibitory effects, indicating its potential application in diabetes treatment.

GPs display a multifaceted array of structural types, including glucans, heteroglucans, galactans, heterogalactans, galactoglucomannans, fucoxylomannans, and others. These polysaccharides exhibit a high level of complexity and diversity in their primary structures, as well as a range of biological activities including immunomodulation, anti-tumor, anti-oxidant, and regulating the intestinal flora. The structural characteristics of GPs are closely related to their biological functions, underscoring their significant potential in medicinal value and biomedical research. These results offer a rigorous scientific foundation and novel pathways for further exploration into the pharmacological mechanisms of *Ganoderma*, as well as the creation of functional products derived from this fungus.

#### Structural characteristics of GPs

The comprehensive analysis of polysaccharides from six *Ganoderma* species reveals shared structural characteristics, including β-(1 → 3)-D-glucan backbones with β-(1 → 6) branching, α-(1 → 6)-linked galactose residues, and molecular weights ranging from 2 to 4000 kDa. These polysaccharides predominantly exhibit triple-helical or spherical conformations and consist mainly of glucans, galactoglucans, and heteropolysaccharides.

Notable species-specific features include *G. lucidum’*s predominant β-D-glucans (GLP20, GLPs) with triple-helical conformation, *G. sinense*'s unique GSPB70-S sequence containing N-acetylglucosamine, and *G. leucocontextum*'s distinctive α-D/β-D-glucan ratio (17.5:1.5) in GLP-3. *G. atrum* produces PSG-1-F0.2 with a characteristic (1 → 3)-β-D-Glcp backbone and high glucuronic acid content (15.3%), while *G. applanatum* synthesizes GAP-2 with a unique α/β-mixed backbone structure and diverse monosaccharide composition (Glc:Man:Gal:GlcA:Xyl = 82.0:5.6:4.3:4.2:3.9).

These structural variations reflect evolutionary adaptations among *Ganoderma* species, contributing to the diverse pharmacological activities observed across different polysaccharide types. Further exploration of structure–activity relationships (SARs) is warranted to better understand their biological roles and support the targeted utilization of GPs in medicinal and functional food applications.

## Biological activity of GPs

### The relationships between chemical structure and bioactivity

The structure–activity relationship of GPs demonstrates a significant dependence on various structural features, including monosaccharide composition, glycosidic bond types, molecular weight (Mw), branching patterns, and spatial conformation. Initially, variations in monosaccharide composition have been observed to impact the activity of GPs. Mannose-rich GPs have been found to bind to mannose receptors (MR) on macrophages, which promotes macrophage activation and enhances their phagocytic capabilities [84, 85]. For instance, GPs containing more than 5% mannose have been shown to activate macrophages more effectively, resulting in increased secretion of TNF-α compared to GPs with only 1.6% mannose [59]. Additionally, studies suggest that an increased mannose content also enhances natural killer (NK) cell activation, thereby improving their tumor-killing capabilities [86, 87].

Besides monosaccharide composition, the glycosidic linkage patterns play a crucial role in determining the bioactivity of GPs. Among various glycosidic types, the β-(1 → 3)-D-glucan backbone forms the primary structural basis for the immunomodulatory and antitumor effects of GPs [88]. The incorporation of β-(1 → 6)-D-glucan branches significantly enhances the bioactivity of β-(1 → 3)-D-glucans, suggesting the importance of branching patterns in function optimization [77, 89]. In particular, β-glucans with a branching ratio between 0.2 (1:5 branching) and 0.33 (1:3 branching) are recognized as the most potent immunomodulators, displaying enhanced capacity to regulate immune responses and inhibit tumor growth [90–92].

The Mw and configuration of polysaccharides are critical factors in determining their bioactivity. High Mw β-glucans, particularly those exhibiting triple-helix structures, are well-known for their potent immunomodulatory and antitumor activities [93]. This superior bioactivity is attributed to their complex three-dimensional conformations, which enable more effective binding to binding to pattern recognition receptors (PRRs). For example, the formation of a β-(1 → 3)-glucan triple-helix has been shown to significantly enhance the activity of immune cells, including macrophages [94]. Additionally, high Mw polysaccharides exhibit extended half-lives in vivo, leading to prolonged biological effects. While a high Mw is not an absolute requirement for immunostimulatory activity, its combination with specific structural features can synergistically enhance biological efficacy. Notably, lower molecular weight β-glucans have also demonstrated significant immunostimulatory potential, indicating that the interaction between Mw and structural properties is crucial for determining overall bioactivity [9].

The spatial conformation of β-glucans, which exists in three primary forms—triple-helix, single-helix, and random coil—is determined by the arrangement of sugar residues, Mw, and hydrogen bonding interactions between and within chains [95–97]. While early studies showed inconsistent correlations between β-glucan conformation and immunomodulatory effects, recent evidence strongly supports the superior immunoactivity of triple-helix structures [98–101]. Specifically, these glucans stimulate monocytes and macrophages to release pro-inflammatory cytokines such as IL-6, IL-1β, and TNF-α, thereby enhancing immune responses and inhibiting tumor growth, even with a relatively loose helical configuration. Conversely, single-helix β-glucans, while also immunoactive, exhibit lower stability and reduced efficacy in modulating immune responses and suppressing tumors [88, 102, 103]. Overall, while triple-helix structures demonstrate optimal immunomodulatory and antitumor effects, single-helix and random coil configurations also contribute to specific biological responses, suggesting a sophisticated recognition system within the host for different β-glucan conformations [95, 104].

### Immunomodulation activity

**Fig. 2:**
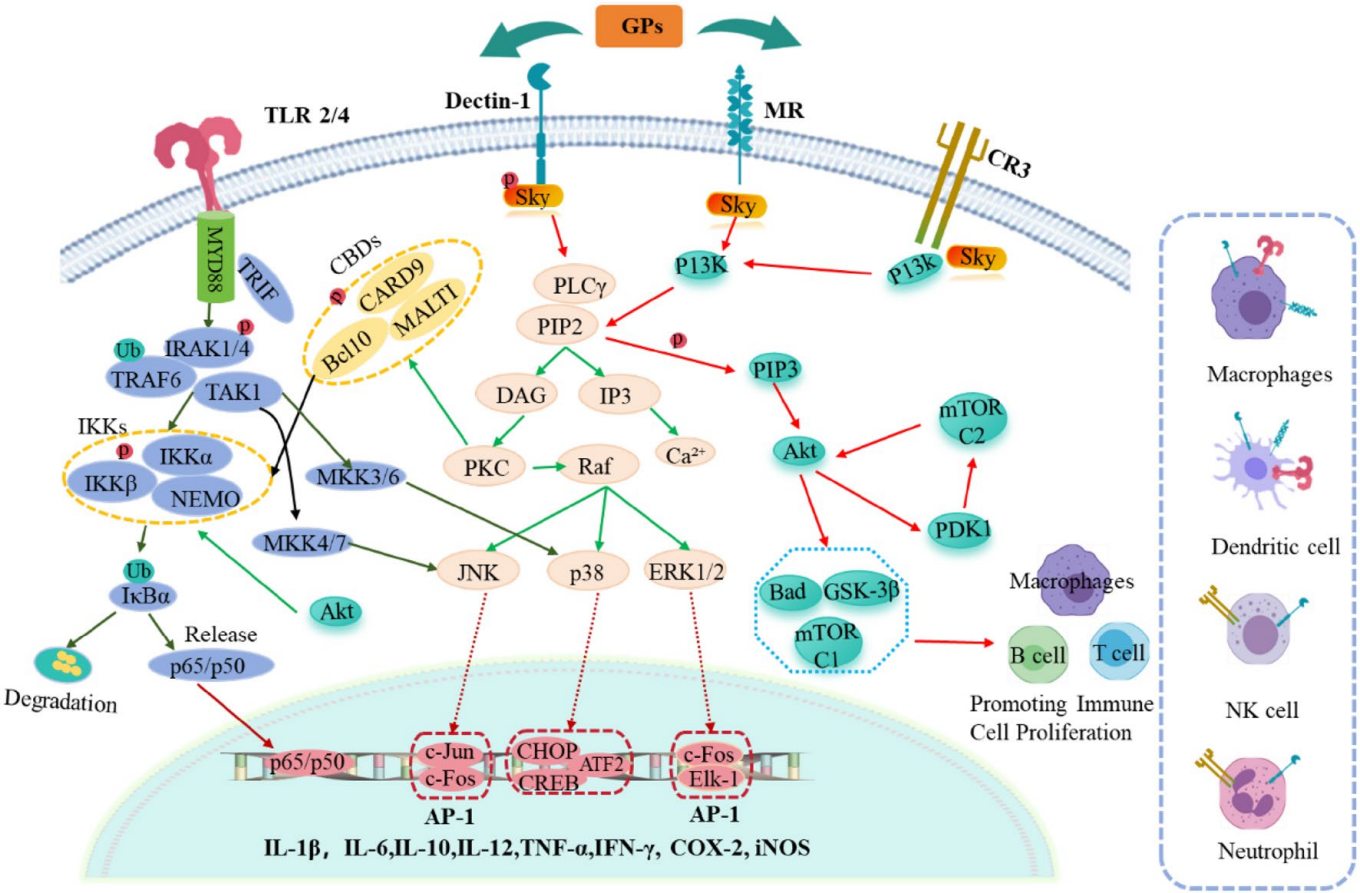
Possible immunomodulatory mechanisms of GPs. GPs interact with key immune receptors, such as Dectin-1, MR, CR3 and TLRs, triggering downstream signaling pathways, including NF-κB, MAPKs, and PI3K/Akt [120]. These pathways lead to the activation of various immune cells, including macrophages, dendritic cells, T cells, B cells, NK cells, and neutrophils. Upon activation, these cells secrete pro-inflammatory cytokines such as TNF-α, IL-1β, IL-6, and IFN-γ, which promote immune cell proliferation, activation, and enhance phagocytosis. These cytokines also inhibit tumor cell proliferation by inducing apoptosis and reducing angiogenesis. For example, Gl-BSP derived from *G. lucidum* significantly increases NK cell and T cell cytotoxicity, contributing to tumor inhibition by upregulating IFN-γ and TNF-α. IFN-γ activates macrophages and NK cells, enhancing their cytotoxicity, while TNF-α induces apoptosis in tumor cells and disrupts the tumor vasculature. In parallel, the PI3K/Akt pathway enhances T cell and B cell survival and proliferation, while mTORC1 activation supports immune cell growth and function. Collectively, these pathways create a robust immune response, modulate the tumor microenvironment, and enhance antitumor immunity

GPs have attracted increasing attention for their multifunctional roles in immune regulation. As shown in Fig. 2 and Table 3, GPs have emerged as a research focal point, given their ability to modulate the immune system through various mechanisms. Studies have demonstrated that GPs bind with high affinity to critical immune receptors, including Dectin-1, mannose receptor (MR), complement receptor (CR), toll-like receptor (TLR) 2, and TLR4, suggesting their potential use in immunomodulatory applications [105].

**Table 3:** Bioactive polysaccharides from six *Ganoderma*

Resources	Structure information	Biological activity	Change	In vitro/in vivo assay	Mechanisms	Dose	References
*G. lucidum*	GLSWA-I	Immunomodulatory activity	Ear swelling ↑	In vivo: Promotes DTH ear swelling	–	75, 150, 300 mg/kg	[63]
*G. lucidum* (fruiting bodies)	GLP20	Immunomodulatory activity	Macrophage activity ↑, NO production ↑	In vitro: RAW264.7 Cells	–	50, 200, 500 µg/mL	[62]
*G. lucidum* (fruiting bodies)	Fucoxylomannan	Antimelanoma activity	Cell viability ↓, cell proliferation ↓, colony formation ↓	In vitro: Reduced melanoma B16-F10 viability	G2/M cell cycle arrest, blocks colony formation	0, 250, 500 μg/mL	[73]
*G. lucidum* (fruiting bodies)	GLP70-1-2	Anti-neuroinflammatory activity	NO ↓, TNF-α ↓, and IL-6 ↓; Inos ↓, COX2, IL-6 ↓, IL-1β ↓, TNF-α ↓, TLR4 ↓, MyD88 ↓, IKKα ↓	In vitro: LPS treated BV2 cell In vivo: AD rats	TLR4/MyD88/NF-κB pathway	4–128 μM (effective at 64 μM)	[76]
Broth of *G. lucidum*	GLP-2 (Galactose-rich polysaccharide)	Immunomodulatory activity	T and B lymphocyte ↑; alanine aminotransferase (ALT) ↓, aspartate aminotransferase (AST) ↓	In vivo: Kun Ming mice	–	25, 75, 150 and 300 mg/kg	[81]
*G. lucidum* (fruiting bodies)	GLPs (β-(1 → 3)-D-glucan)	Anti-inflammation activity	NO ↓, TNF-α ↓, iNOS ↓	In vitro: LPS treated RAW 264.7 cell	NF-κB Pathway (IκB-α↑), MAPKs (p-JNK↓, p-ERK↓, p-p38↓)	20, 50 and 100 μg/mL	[77]
*G. lucidum* (spore)	GLSP-I	Immunomodulatory activity	NO ↑, macrophage activity ↑	In vitro: RAW264.7 Cells	–	5–200 μg/mL	[61]
*G. lucidum*	LZ-D-1	Immunomodulatory activity	Mouse spleen lymphocytes ↑	In vitro: Mouse spleen lymphocytes	–	50, 200 and 500 μg/mL	[69]
*G. lucidum*	GLSP (β-(1 → 3),(1 → 6)-D-glucan)	Antitumor activity	Tumor inhibition ↑, cytokine levels ↑	In vivo: Sarcoma S180, Lewis lung cancer, liver cancer H22, and colon cancer C26 mouse models	PI3K/Akt pathway	12.5–50 mg/kg	[78]
*G. lucidum*	GLP-1 Man:Glc:Gal:Fuc (4.9:63.5:26.2:5.4); GLP-2: β-D-glucan with glucose (90.6%)	Immunomodulatory activity	IgA ↑, IgG ↑, spleen/thymus indices ↑	In vivo: Restored immune indices in cyclophosphamide-treated mice	–	250 mg/kg	[59]
*G. lucidum* (spores)	WGLP	Antitumor activity	Tumor weight ↓	In vivo: S180-bearing mice	–	3–100 mg/kg	[80]
*G. lucidum*	GLP: β-(1 → 3)-D-glucan with branched β-(1 → 6)-D-glucopyranosyl residues	Prebiotic: improves gut microbiota composition, increases beneficial bacteria, and enhances intestinal barrier functions	SIgA ↑, IL-2 ↑, IL-4 ↑, DAO ↓	In vivo: Male SD rats	Activates NF-κB and upregulates occludin	100 mg/kg	[148]
*G. lucidum*	GLP: Heteropolysaccharide mainly composed of glucose (75%)	Prebiotic: modulates gut microbiota, improves SCFA production, and reduces inflammation	Gut microbiota ↑ (*Lactobacillus*, *Bacteroides*), SCFA ↑ (acetate, butyrate), IL-6 ↓, TNF-α ↓	In vivo: Male C57BL/6 mice	Inhibits NF-κB pathway; enhances intestinal barrier proteins (ZO-1, occludin)		[146]
*G. sinense*	GSPB70-S	Immunomodulatory, antioxidant, and hypoglycemic activity	NO ↑, α-glucosidase inhibition ↑	In vitro: RAW264.7 macrophages	–	250–500 μg/mL	[74]
*G. sinense* (fruiting bodies	GSBP-2	Anti-breast cancer migration and invasion	Inhibit the proliferation, migration, and invasion of MDA-MB-231 cells	In vitro: MCF-7 and MDA-MB-231 cells	PI3K/Akt pathway	50–200 µM	[82]
*G. sinense*	GSP-2	Immunomodulatory activity	TNFα, IL1β, IL6 secretion ↑	In vitro: RAW264.7 macrophages	Acts on TLR4	12.5–100 μg/ml	[149]
G. sinense and *G. lucidum*	structural similar in GS and GL	Antitumor, immunomodulatory, gut microbiota modulatory	Phagocytic activity ↑; NO, IL-6, TNF-α production ↑; Firmicutes ↓, Bacteroidetes ↑ GL and GS exhibit significant bioactivity with GS demonstrating slightly stronger effects than GL	In vivo: 4T1 breast cancer-bearing mice	Activation of MAPKs and NF-κB signaling pathways	200 mg/kg/day	[150]
*G. atrum*	PSG-1:Homogeneous protein-bound polysaccharide, composed mainly of mannose, galactose, and glucose in a molar ratio of 1:1.28:4.91; PSG-1-F0.2: Highly branched acidic β-(1 → 3, 1 → 6)-glucan, containing Glc:GlcA:Man at area (%) ratio of 73.8:15.3:5.7:5.2	Immunomodulatory activity	Macrophages: (phagocytosis, NO, TNF-α, IL-1β, ROS) ↑; Lymphocytes: ↑ Ca^2+^ → ↑ CaN activity → ↑ NFAT, IL-2 → PKC activation; Dendritic Cells (DCs): ↑ Maturation/activation → ↑ MHC-II, CD80, CD86 → ↑ Cytokines (IL-12, IL-6, IL-10, RANTES, MIP-1a, MCP-1); Protective Effects: ↓ Acrolein-induced apoptosis, ↓ ROS, ↑ MMP, regulates Bcl-2, Cyt-C, caspase-3, caspase-9	In vitro: RAW264.7 cells. In vivo: C3H/HeN mice;	Activation of TLR4/ROS/PI3K/Akt/MAPKs/NF-κB; Activation of the Ca2 + /CaN/NFAT/IL-2 and PKC/NFAT/IL-2; Activation of the mTOR signaling pathway	20, 40, 80, 160 mg/mL	[151–156]
		Antitumor activity	Suppresses tumor growth in CT26-bearing mice	In vitro: CT26 cells, In vivo: CT26-bearing mice	Activates the MAPK, NF-κB, and cAMP-PKA signaling pathways	50, 100, 200 mg/kg	[157, 158]
		Hypoglycemic activity	Endothelial nitric oxide synthase (eNOS) activity ↑, reduces fasting blood glucose (FBG) ↓, improves endothelium-dependent aortic relaxation, decreases apoptosis of endothelial cells	In vitro: Endothelial cell apoptosis assay, In vivo: Diabetic rat model	Activation of PI3K/Akt/eNOS pathway, upregulation of Bcl-2, downregulation of Bax	200, 400 mg/kg	[159]
		Protective effect on colitis	Goblet cells and tight junction proteins ↑; Bcl-2 inhibition, caspase-3/9 ↓; Atg5, Atg7, beclin-1 ↑; p-akt, p-mTOR ↓; DC content ↓, modulates IL-10 in DCs	In vivo: DSS-induced colitis model in mice	Enhances autophagy via the Akt/mTOR pathway, modulates DC-related immune responses	50, 100, 200 mg/kg	[160, 161]
		Anti-inflammatory activity	Tight junction (TJ) proteins ↑, mitochondrial membrane potential (MMP) ↑, B-cell lymphoma 2 Bcl-2 ↑, caspase-3/9 ↓	In vivo: Mice model with acrylamide-induced intestinal damage	NF-κB signaling pathway, enhances tight junction protein expression (Occludin, Claudin-1, ZO-1)	50, 100, 200 mg/kg	[72]
*G. tsugae*	GTWE	Anti-invasive and pro-apoptotic effects on metastatic melanoma cells	ROS production ↓, melanoma cell migration ↓, viability ↓, BCL2 expression ↓, Bax ↑, cleaved caspase-9 ↑	In vitro: B16F10 and LMM cell lines; In vivo: Lung metastatic melanoma mouse model	PI3K/Akt signaling pathway	5–125 μg/mL (in vitro); 2–10 mg/mL (in vivo)	[162]
*G. applanatum*	GAP	Anti-tumor and anti-inflammatory activities	Tumor volume ↓, TAMs (CD68, CD163) ↓, Arg-1 ↓, TGF-β ↓, Ki67 ↓, CD31 ↓	In vitro: RAW264.7 cells; In vivo: Rabbit VX2 liver tumor model	–	15 μg/mL (in vitro); 200 μg/kg (in vivo)	[163]
*G. applanatum*	GAP	Protective effect against DSS-induced colitis	Colon length ↑; COX-2, iNOS, MPO activities ↓; Intestinal barrier proteins ↑	In vivo: DSS-induced colitis in mice	Modulation of gut microbiota, enhancement of intestinal barrier↑ (ZO-1, MUC2, claudin-3, occludin)	250, 500 mg/kg	[164]
*G. applanatum*	GAP	Anti-tumor activity in MCF-7 breast cancer cells	Proliferation ↓, apoptosis ↑, migration ↓ in MCF-7 cells; autophagy ↑, p-ERK ↓, p-p38 ↑, p-JNK ↑	In vitro: MCF-7 cells	MAPK signaling pathway	125, 250, 500 μg/mL	[165]
*G. applanatum*	GAP-3S	Anti-tumor activity in MCF-7 cells	Cell proliferation ↓, apoptosis ↑, ROS generation ↑, MMP collapse, Bax ↑, Bcl-2 ↓, PARP cleavage ↑	In vitro: MCF-7 cells	MAPK signaling (p38 ↑, JNK ↑, ERK ↓)	125, 250, 500 μg/mL	[166]
*G. applanatum*	GRP with Mw 12.2 kDa, Rha:Fuc:Man (1.99:1.21:6.33:6.78)	Hepatoprotective and anti-inflammatory activity	ALT, AST, and ALP levels ↓; SOD, GSH-Px, and CAT activities ↑; TNF-α, IL-6 ↓; IL-10 ↑	In vivo: CCl_4_-induced liver injury in mice	NF-κB signaling pathway	100, 200, 400 mg/kg	[167]
*G. applanatum*	GLP-1	Immunostimulatory activity	Phagocytic activity ↑, ROS production ↑, NO production ↑, TNF-α, IL-6 ↑	In vitro: RAW264.7 macrophages; In vivo: CTX-induced immunosuppressed mice	Activation of MAPKs, PI3K/Akt, and NF-κB signaling pathways	40, 80, 160 mg/kg	[168]
*G. applanatum*	GLP-1 and GLP-2	Antioxidant activity	MDA ↓, CAT ↑, GSH-Px ↑, GSH/GSSG	In vitro: NIH3T3 cells	–	25, 50, 100 µg/mL	[169]
*G. leucocontextum*	GLP-3 with molecular weight of 159.7 kDa	Immunomodulatory activity	Pinocytic capacity ↑, Phagocytic capacity ↑, NO, TNF-α, IL-6, IL-1α, IL-1β, IL-10, CXCL5, MIP-2, MCP-1 secretion ↑	In vitro: RAW264.7 macrophages	Activation of MAPKs, PI3K/Akt, and NF-κB signaling pathways	25, 50, 100 µg/mL	[170]

The upward arrow “↑” in the sequence represents an “increase” or “elevation” in the level or activity of the specified molecule or process; The arrow “ → ” in the sequence represents “leads to” or “results in.”

Dectin-1, a C-type lectin-like receptor expressed on immune cells such as macrophages, dendritic cells, neutrophils, and monocytes, plays a pivotal role in recognizing and binding β-glucans with β-(1,3) and/or β-(1,6) glycosidic linkages [106, 107]. Upon activation of Dectin-1, downstream Syk kinase undergoes phosphorylation, subsequently activating PLCγ, which subsequently triggers the activation of protein kinase C (PKC) and the production of reactive oxygen species (ROS) [108]. These signaling events lead to the activation of two key pathways. The first involves the activation of PKC, which triggers the Card9/Bcl10/Malt1 complex, promoting the downstream activation of NF-κB and MAPK pathways, driving the production of pro-inflammatory cytokines [109, 110]. The second pathway involves the generation of ROS, which acts as a signaling molecule to further enhance immune responses by activating the inflammasome, leading to the maturation of cytokines such as IL-1β and amplifying the inflammatory response [111].

Recent studies identified a β-1,3-D-glucan (GSG) derived from *Ganoderma* spores, characterized by a β-1,3-glucan backbone with β-1,6-glucose side chains. GSG binds to Dectin-1 and activates the MAPK signaling pathway, resulting in significant immunomodulatory effects [112]. Other polysaccharides with similar structural characteristics also enhance the activation and maturation of immune cells via pattern recognition receptors, including Dectin-1, scavenger receptors, CR3, and TLR4. These interactions activate downstream signaling molecules such as Syk, JNK, p38, ERK, and NF-κB [113–115]. Furthermore, GLP-3, a water-soluble polysaccharide isolated from *G. leucocontextum*, with a molecular weight of 159.7 kDa and an α-D-1,4-glucose backbone, exhibits notable immunomodulatory activity [57]. GLP-3 enhances macrophage phagocytosis and pinocytosis while promoting cytokine production through TLR2-mediated activation of MAPKs (JNK, ERK, and p38), PI3K/Akt, and NF-κB pathways.

GPs exhibit remarkable immunomodulatory potential by engaging with critical immune receptors and activating essential signaling pathways, such as NF-κB, MAPK, and PI3K. These mechanisms highlight the potential of GPs as promising therapeutic candidates for immune regulation. Future studies should explore their clinical applications and potential synergistic effects with existing immunotherapies to address inflammatory and autoimmune diseases.

### Immune-mediated antitumor activities

GPs activate immune cells by PRRs on the surface of immune cells, enhancing their phagocytic and stimulating the secretion of pro-inflammatory cytokines including TNF-α, IL-1, and IL-6. These cytokines inhibit tumor cell proliferation and promote apoptosis while modulating the tumor microenvironment (TME) by facilitating the recruitment and activation of additional immune effector cells, thus enhancing overall antitumor immunity.

Gl-BSP, derived from *G. lucidum* broken spore, exerts significant antitumor effects by enhancing the activity of NK cells, T cells, and macrophages [116]. The results showed that neutralization with antibodies against TNF-α and IFN-γ significantly reduced the tumor-inhibitory effects of Gl-BSP on S180 and PG tumor cells. Similarly, GL-PS has been shown to inhibit glioma growth by increasing serum IL-2, TNF-α, and IFN-γ, which enhances the cytotoxic activity of NK cells and T cells and promotes dendritic cell maturation [117]. SBSGL downregulated PD-1/PD-L1, enhancing Th1 immune responses, with increased TNF-α and IL-2 and reduced IL-10 and IL-6 levels, indicating stronger antitumor immunity and suppression of Th2 responses [118]. Additionally, GLPs enhance CD8^+^T cell secretion of IFN-γ and perforin, strengthening antitumor immunity in the tumor microenvironment [119]. Furthermore, combining GLP with anti-PD-1 antibodies significantly improves the efficacy of anti-PD-1 immunotherapy, underscoring its potential to boost immunotherapeutic outcomes.

In summary, GPs exert significant immunomodulatory and immune-mediated antitumor activities by influencing immune-related cells such as B cells, T cells, DCs, macrophages, and NK cells. Their antitumor effects are primarily mediated through immunoregulation, anti-angiogenesis, and cytotoxic mechanisms. These findings suggest the presence of a complex biological system within the host capable of recognizing and responding to different glucan structures, offering new perspectives for further exploration and the development of functional food and therapeutic applications.

### Non-immune antitumor activities

**Fig. 3:**
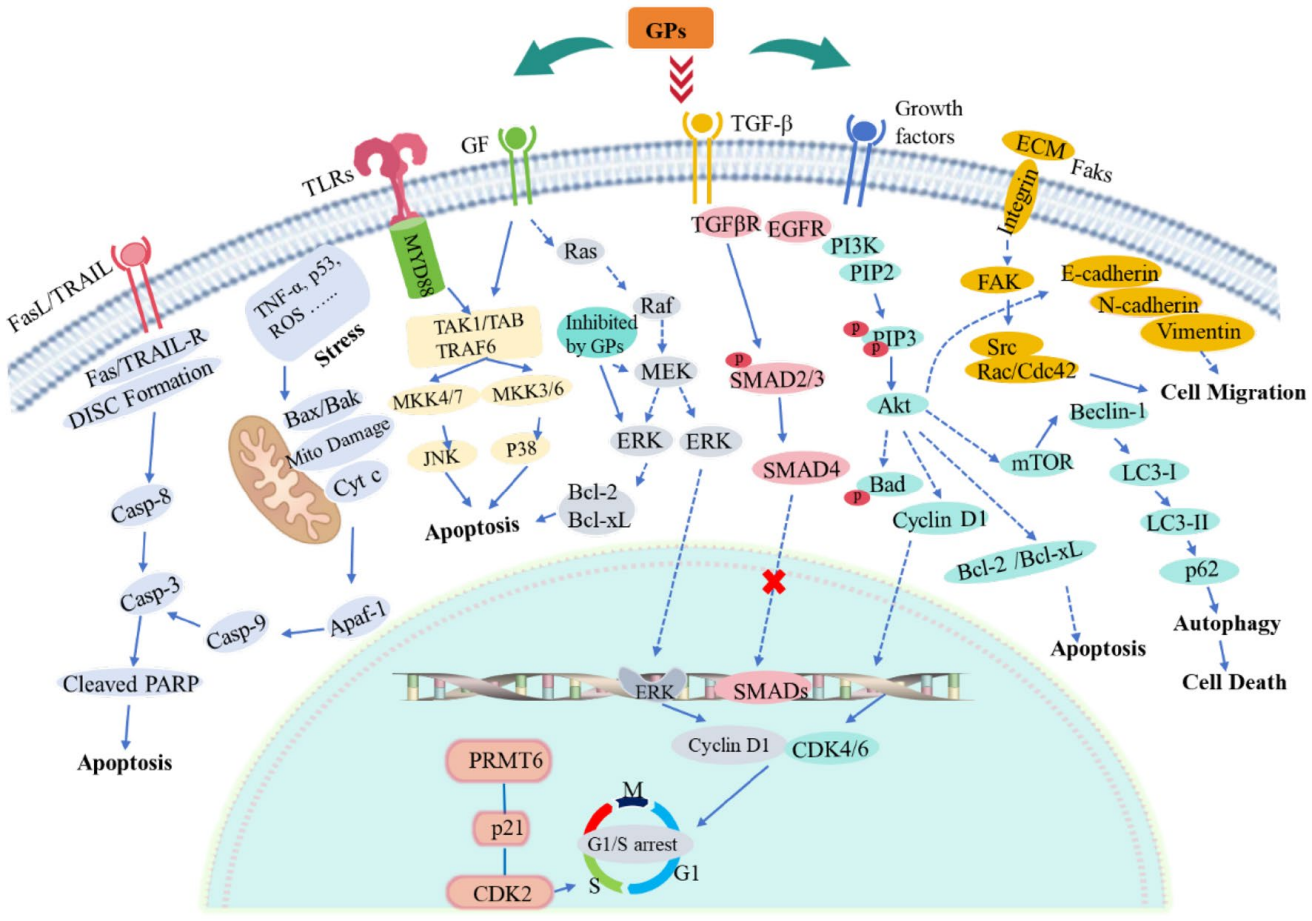
The antitumor mechanisms of GPs via multiple signaling pathways. 1. Mitochondrial apoptosis pathway: GPs disrupt mitochondrial membrane potential, leading to cytochrome c release, which activates downstream caspase-9 and caspase-3, ultimately resulting in apoptosis. 2. Death receptor pathway: GPs upregulate death receptors such as Fas and TRAIL-R, along with their ligands, activating the extrinsic apoptotic pathway. This leads to the formation of the death-inducing signaling complex (DISC) and activates caspase-8 and caspase-3, which subsequently induce apoptosis. GPs promote apoptosis in colon cancer cells such as HCT-116 and LoVo through this Fas-mediated, caspase-dependent pathway. 4. FAK/PI3K/Akt and MAPK pathways: GPs inhibit the PI3K/Akt pathway, which downregulates anti-apoptotic proteins such as Bcl-2 and Bcl-xL, promoting apoptosis. Additionally, GPs obstruct cancer cell proliferation and migration by inhibiting the FAK/Src/Rac/Cdc42 pathway, which is essential for cell motility. GPs also regulate the MAPK pathway, inhibiting ERK signaling, which suppresses cell proliferation. 5. Autophagy Modulation: GPs regulate autophagy by inhibiting the Akt/mTOR pathway, resulting in the activation of autophagy-related proteins, including Beclin-1, LC3-I, and LC3-II, and promoting p62 degradation. This modulation leads to enhanced autophagy, which culminates in cancer cell death. Compounds such as RSGLP increase autophagosome accumulation and disrupt autophagic flux, contributing to the inhibition of cancer cell proliferation. 6. EMT Inhibition: GPs suppress the EMT by upregulating E-cadherin and downregulating mesenchymal markers such as N-cadherin and Vimentin. This reduces tumor cell migration and invasion. The polysaccharide GSBP-2 inhibits EMT by downregulating mesenchymal markers and blocking the PI3K/Akt pathway, further inhibiting cancer cell metastasis. 7. Cell Cycle Regulation: GPs induce cell cycle arrest by upregulating p21, which suppresses the activity of CDK2 and Cyclin D1, leading to a halt at the G1/S transition. GPs also downregulate PRMT6, reducing the activity of CDK2, FAK, and SRC, which enhances cell cycle arrest and promotes apoptosis. This mechanism is crucial for inhibiting tumor cell proliferation

GPs demonstrate significant antitumor activities not only by enhancing host immune responses but also through direct effects on tumor cells via multiple mechanisms [89, 121]. As shown in Fig. 3 and Table 3**,** these mechanisms include: (1) apoptosis induction through both mitochondrial pathway (disrupting mitochondrial membrane potential, promoting cytochrome c release, and activating caspase-3/caspase-9 cascade) and death receptor pathway (upregulating Fas/TRAIL-R expression and activating caspase-8-dependent pathway), ultimately leading to PARP cleavage and programmed cell death; (2) proliferation and metastasis inhibition through regulation of cell cycle proteins (p21, CDK2) and key signaling pathways (PI3K/Akt, MAPK, FAK), as well as EMT-related proteins (E-cadherin, N-cadherin, Vimentin, Snail1, and ZEB1) and growth factor receptors (EGFR, TGFβR); and (3) autophagy modulation via regulation of key proteins (LC3-II, p62, RACK1) and disruption of autophagosome-lysosome fusion, affecting cellular stress responses and survival. These molecular mechanisms work synergistically at multiple cellular levels to achieve comprehensive antitumor effects.

The apoptotic effects of GPs are primarily mediated through both intrinsic and extrinsic pathways, which converge at the activation of caspase-3, thereby amplifying the apoptotic response. SeGLP-2B-1, a selenium-enriched heteropolysaccharide (1.06 × 10⁶ Da) with a β-1,3-glucan backbone and β-1,6-glucan side chains, demonstrates this mechanism by inducing mitochondrial membrane potential disruption and triggering the caspase cascade activation [122, 123]. Similarly, BSGLWE from *Ganoderma* spores induces apoptosis by modulating Bcl-2 levels and activating caspase-3/9 [124, 125]. The combination of GLPs (Mw > 10 kDa) with 5-fluorouracil further demonstrates this mechanism by reactivating mutant p53 and enhancing mitochondria-mediated apoptosis [126], while crude GLPs induce Fas-mediated apoptosis in colon cancer cells [127, 128].

GPs exhibit potent anti-proliferative and anti-metastatic effects through modulation of multiple interconnected signaling pathways. GAP-2 (21.3 kDa) from *G. lucidum* significantly inhibits various cancer cell lines including A549, SKOV3, and SMMC-7721, with a survival rate of 60.9% in A549 cells at 600 μg/mL [65]. WSG, a water-soluble glucan with an Mw of approximately 1000 kDa, suppresses cancer cell growth and migration by inhibiting ERK, AKT, FAK, and TGFβR signaling pathways [129–131]. The complex polysaccharide GLP, composed of Ara, Gal, Glc, and Xyl in a molar ratio of 4:2:10:1, arrests prostate cancer cell cycle through PRMT6 pathway regulation [132, 133]. GSBP-2 (11.5 kDa) prevents breast cancer metastasis by inhibiting EMT through PI3K/Akt pathway modulation and regulating EMT-related proteins including E-cadherin, N-cadherin, Vimentin, Snail1, and ZEB1 [82].

The autophagy-regulating effects of GPs represent another crucial mechanism in their antitumor activity, functioning as both a tumor suppressor and a modulator of apoptotic responses. RSGLP, at a concentration of 200 µg/ml, effectively disrupts autophagic flux by modulating LC3-II and p62 expression, leading to autophagosome accumulation and subsequent cancer cell death [134]. SBSGL, isolated from *G. lucidum* spore broken powder, demonstrates similar effects by inhibiting hepatoblastoma progression through RACK1-mediated autophagy regulation, specifically by reducing RACK1 protein expression through O-GlcNAc modification inhibition, disrupting autophagosome-lysosome fusion, and modulating the LC3-II/LC3-I ratio [118].

This comprehensive antitumor activity of GPs, encompassing apoptosis induction, proliferation inhibition, and autophagy regulation, along with their well-characterized molecular mechanisms, positions them as promising candidates for cancer therapy. The diverse mechanisms through which GPs exert their antitumor effects suggest potential advantages in targeting multiple aspects of tumor development and progression simultaneously.

### Regulate the gut microbiota

**Fig. 4:**
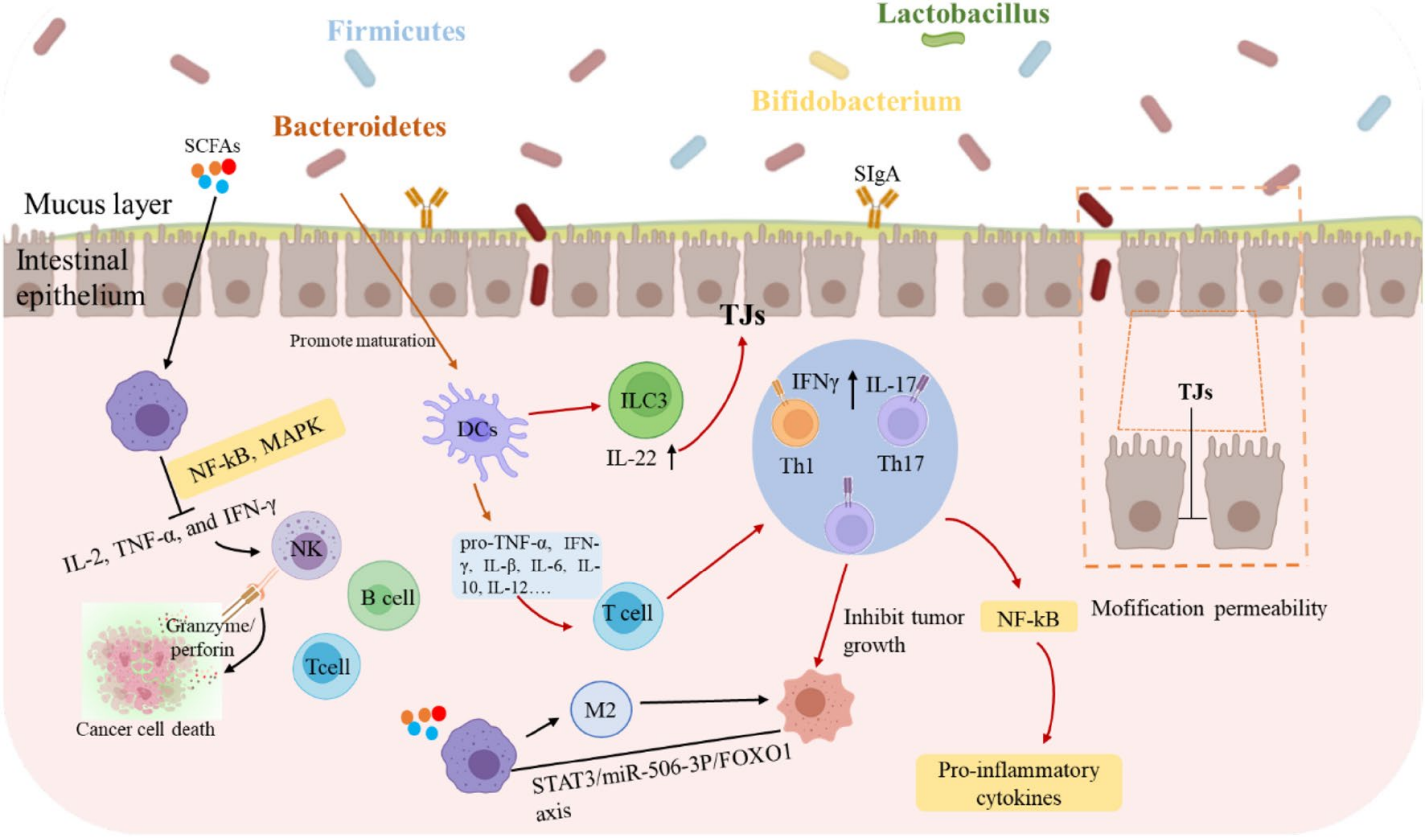
Regulatory Effects of GPs on gut microbiota and immune system: implications for tumor suppression and intestinal health [11]

GPs regulate intestinal homeostasis through multiple mechanisms and pathways [11]. As shown in Fig. 4 and Table 3, these mechanisms include: (1) enhancement of intestinal barrier function through upregulation of tight junction proteins and modulation of signaling pathways; (2) modulation of gut microbiota composition and metabolite production, particularly SCFAs; and (3) regulation of immune responses, inflammatory processes, and anti-tumor through multiple pathways, ultimately leading to improved intestinal health.

The complex structures of GPs render them impervious to direct digestion and absorption by the digestive system of the host, facilitating their transit to the colon. During simulated gastric and intestinal digestion, GPs exhibited remarkable stability with molecular weight only decreasing from 198.0 kDa to 147.1 kDa [135]. In the colon, polysaccharides from *Ganoderma* mycelium exemplify this mechanism by strengthening multiple barrier components: mechanical barrier (upregulating occludin), immune barrier (increasing IL-2, IL-4, and SIgA levels), and biological barrier function [136]. This protective effect is particularly evident in chemotherapy-induced intestinal injury, where co-administration of homogeneous GPs (SGP, Mw 3.6 kDa) with paclitaxel effectively mitigates barrier damage through upregulation of tight junction proteins (TJs) including ZO-1, E-cadherin, β-catenin, and occluding [137–139]. Additionally, studies have shown that GPs may enhance tight junction dynamics by modulating signaling pathways such as PI3K/Akt /mTOR and NF-κB, both of which play key roles in regulating the expression of TJs [138, 139].

GPs exhibit potent modulatory effects on gut microbiota composition and metabolite production. In the colon, GPs significantly modulate gut microbiota by selectively promoting beneficial bacteria (*Bifidobacteria* and *Lactobacilli*) while suppressing pathogenic species (such as *E. coli* and *Clostridium perfringens*). *G. lucidum* spore polysaccharides (CPGS and RPGS) demonstrate distinct effects on microbial composition, with CPGS increasing *Verrucomicrobia* and *Proteobacteria*, while RPGS enhances *Actinobacteria* populations, both significantly enriching immune-regulatory bacterial genera, including *Adlercreutzia*, *Prevotella*, and *unclassified Desulfovibrionaceae* [136]. These beneficial bacteria efficiently utilize GPs, particularly β-D-glucan-rich heteropolysaccharides, to produce short-chain fatty acids (SCFAs), including acetate, propionate, and butyrate, through microbial fermentation [140, 141].

The immune-modulatory and anti-inflammatory effects of GPs are mediated through multiple mechanisms. SCFAs, especially butyrate, exhibit significant anti-inflammatory effects through dual mechanisms: activation of G-protein coupled receptors (GPR43) and inhibition of histone deacetylases (HDACs) [142]. This leads to reduced production of pro-inflammatory cytokines such as IL-6 and IL-8 [143], and decreased IL-23 expression in intestinal epithelial cells through reduced STAT1 levels [144]. Additionally, β-D-glucans from *Ganoderma* effectively restore Th17/Treg cell balance, while GLPs demonstrate significant effects in inhibiting colitis and tumor development through modulation of immune cell function [145]. The modulatory effects of gut microbiota on immune regulation and tumor development are further evidenced by specific bacterial populations. Studies have shown that increased abundance of *Alistipes* is associated with tumor growth inhibition [146]. Moreover, Spearman correlation analysis revealed a significant negative correlation between *Ruminococcus* abundance and fructose-6-phosphate levels in tumors, suggesting a close relationship between gut microbiota modulation and tumor metabolism [147].

These findings demonstrate the complex interplay between GPs-mediated immune regulation and gut microbiota modulation, where SCFAs serve as key mediators in anti-inflammatory responses, while specific bacterial populations contribute to both immune homeostasis and tumor suppression through distinct metabolic pathways.

## The biosynthesis of GPs

The cell wall of *G. lucidum* is primarily composed of various polysaccharides, which play a critical role in maintaining the structural integrity of the cell wall and conferring unique biological activities. The basic framework of the cell wall is made up of chitin, β-glucans, and α-glucans [171–174]. The biosynthesis of these polysaccharides is a multi-stage process involving the cytoplasm, endoplasmic reticulum, and Golgi apparatus [10]. During this process, nucleotide sugar precursors are polymerized into short chains, which are subsequently elongated into long chains and modified by branching. These polysaccharides are then transported to the cell wall via ABC transporter-dependent and Wzy-dependent pathways, where they are assembled into a complete structure [175]. The polysaccharide-rich cell wall not only provides physical robustness but also imparts a variety of functional properties, enabling *Ganoderma* to exhibit significant immunomodulatory, anti-tumor, and regulate gut microbiota.

### Biosynthesis of the nucleotide sugar

Nucleotide sugars are indispensable precursors in the biosynthesis of complex polysaccharides, such as glucans, mannans, and galactofucans, which are essential components of fungal and plant cell walls. These nucleotide sugars act as activated donors of sugar residues, facilitating the elongation of polysaccharide chains through the action of glycosyltransferases. While the nucleotide sugar biosynthesis pathways are relatively well-defined and conserved across various organisms, the biosynthesis of GPs exhibits unique regulatory mechanisms and enzymatic specificity.

In GPs production, several enzymes involved in phosphosugar metabolism and glycosyltransferases play pivotal roles. Key enzymes such as phosphoglucomutase (PGM) and phosphomannomutase (PMM) are critical in the production of nucleotide sugars like UDP-glucose and GDP-mannose, which are essential for polysaccharide biosynthesis [176]. These enzymes not only influence intracellular and extracellular polysaccharide yields but also affect the structural composition of the polysaccharides. For instance, silencing of PGM reduces extracellular polysaccharide (EPS) production while increasing intracellular polysaccharide (IPS) levels, indicating its significant role in modulating polysaccharide distribution [177, 178]. Similar studies found that overexpression of PGM resulted in a 40–44% increase in polysaccharide yield and significant upregulation of related genes [179].

Transcriptional regulation also plays a crucial role in nucleotide sugar biosynthesis in *Ganoderma*. The transcription factor GlbHLH has been shown to regulate the expression of key genes involved in polysaccharide synthesis, such as PGM and UDP-glucose pyrophosphorylase (UGP) [180]. Silencing GlbHLH results in reduced polysaccharide production and altered cell wall composition, highlighting its importance in regulating the biosynthesis of nucleotide sugar precursors. Overexpression of GlbHLH or related genes significantly enhances polysaccharide yield, particularly through the upregulation of glycosyltransferase activity.

Genetic modifications, such as the overexpression of PMM1 or the introduction of heterologous genes like VHb, have been successfully employed to increase polysaccharide production in *Ganoderma* [181]. For example, overexpression of PMM1 resulted in a 1.41-fold increase in extracellular polysaccharide production, with a corresponding increase in mannose content, which enhanced the immunomodulatory properties of the polysaccharides [182]. Furthermore, the use of symbiotic fungal inducers has been shown to activate biosynthetic pathway genes, leading to a 3.4-fold increase in polysaccharide production and significant alterations in sugar composition, particularly a decrease in glucose content and an increase in mannose, galactose, and other sugars [183].

In summary, nucleotide sugar biosynthesis in *Ganoderma* is regulated by both enzymatic and transcriptional mechanisms. Through genetic engineering and biological induction strategies, both polysaccharide yields and their bioactivity have been significantly enhanced, providing valuable approaches for industrial GPs production and applications.

### GPs elongation, modification, and the role of enzymes

The biosynthesis of polysaccharides in *Ganoderma* is a complex process involving the elongation, branching, and modification of polysaccharide chains, primarily facilitated by glycosyltransferases (GTs) and glycoside hydrolases (GHs). GTs are responsible for recognizing and transferring activated nucleotide sugars to acceptor molecules, leading to the gradual elongation of polysaccharide chains [184, 185]. Genomic studies have identified 16 nucleotide sugar biosynthesis enzymes and 80 GT-encoding genes in *G. lucidum* mycelium, highlighting the diverse range of enzymes involved in polysaccharide biosynthesis [186]. Among these enzymes, the synthesis of β-(1,3)-glucan is performed by a plasma membrane-bound glucan synthase complex that uses UDP-glucose as a substrate to polymerize glucose monomers into β-(1,3)-glucan chains [187]. These chains are extruded through the plasma membrane and integrated into the cell wall matrix, where they undergo cross-linking and modification by transglycosylases, forming a stable network structure. The glucan synthase complex consists of two key proteins: a catalytic subunit encoded by the FKS/GSC gene, responsible for polysaccharide chain synthesis, and a regulatory subunit encoded by the RHO1 gene, which controls glucan synthase activity by cycling between inactive GDP-bound and active GTP-bound states [188]. Other polysaccharides, such as xylomannans and fucogalactans, are synthesized through pathways regulated by key genes identified via genomic sequence comparison and functional annotation, including Och1p, Van1p, Anp1p, and Mnn9p, all of which are potentially involved in the synthesis of these complex heteropolysaccharides [189].

In addition, proteins with the SKN1 domain play crucial roles in the biosynthesis of β-(1,6)-glucans, which are essential for the branching and structural integrity of fungal cell walls [190]. These proteins have been well-characterized in *Saccharomyces cerevisiae*, where they contribute to the formation of branched glucan structures that enhance cell wall flexibility and strength [191]. GHs play a pivotal role in the hydrolysis and remodeling of polysaccharides, including branch formation and side chain elongation. For example, The GH72 family of β-1,3-glucan transferases extends β-1,6-glucan chains by incorporating branches, which are essential for enhancing immunomodulatory and antitumor activities [176]. GH16 and GH17 families further support cell wall integrity by cross-linking β-glucans and chitin [192, 193]. In *Saccharomyces cerevisiae* and *Aspergillus fumigatus*, GH16 enzymes (e.g., Crh1p, Crh2p) and GH17 enzymes (e.g., Bgl2p) facilitate β-1,6-glucan and chitin cross-linking, maintaining cell wall rigidity and flexibility [194, 195]. Specifically, Bgl2p cleaves and transfers β-1,3-glucan in *S. cerevisiae*, forming curved polymers, while AfBgt1p and AfBgt2p in *A. fumigatus* generate curved and branched glucans, enhancing cell wall complexity.

**Fig. 5:**
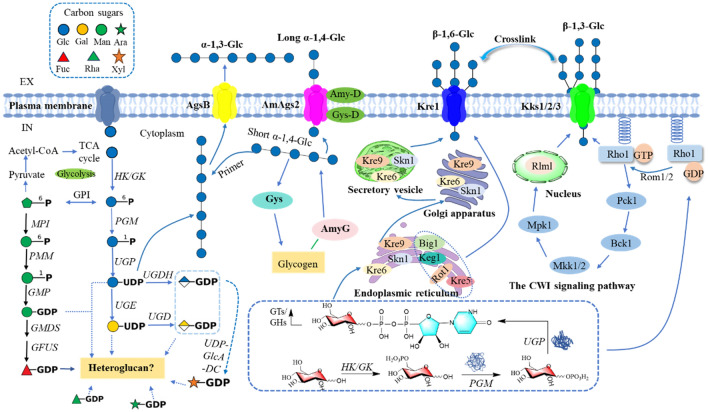
Biosynthesis of polysaccharides in *Ganoderma* [176]*.* The biosynthesis of homopolysaccharides (e.g., β-(1,3)-glucans, β-(1,6)-glucans, α-glucans) and heteropolysaccharides (e.g., glucomannans, galactofucans) in *Ganoderma* begins with monosaccharide uptake, followed by phosphorylation and conversion into nucleotide sugars including UDP-glucose, GDP-mannose, and UDP-glucuronic acid. GTs elongate these nucleotide sugars into complex polysaccharides, which are subsequently modified by transglycosylases and glycoside hydrolases (GHs). The glucan synthase complex (FKS and RHO1) plays a pivotal role in β-(1,3)-glucan synthesis. This intricate assembly enhances cell wall integrity and contributes to the immunomodulatory, antioxidant, and antitumor activities of *Ganoderma*

As shwon in Fig. 5, polysaccharide elongation, modification, and structural assembly in *Ganoderma* are facilitated by a diverse set of glycosyltransferases and glycoside hydrolases. These enzymes work in tandem to create complex, branched polysaccharides that form the fungal cell wall, with GTs responsible for polymerization and GHs for modification and cross-linking. Understanding these enzymatic processes provides key insights into the regulation of polysaccharide biosynthesis and the potential for applications in functional food development.

## Functional food applications of GPs

GPs, with their diverse bioactive properties, have demonstrated significant potential in the functional food industry. Comprehensive reviews emphasize that their immune-modulating, anti-tumor, and prebiotic characteristics have led to extensive commercial applications in this field. GPs are widely incorporated into functional beverages, such as herbal teas and energy drinks, especially in the Asian market, where their immune-boosting and fatigue-reducing effects are highly regarded [196]. These products capitalize on GPs’ capacity to activate macrophages, natural killer (NK) cells, and T cells, providing a natural means of supporting immune health [197]. Additionally, GPs hold a prominent place in the global dietary supplement market, especially in the US and Europe. Here, they are formulated into capsules, tablets, or powders, marketed for their immune-regulatory, anti-inflammatory, and tumor-suppressing effects [146]. Further expanding their application, GPs are increasingly integrated into functional snacks and food additives, such as energy bars and cereals, often in combination with probiotics and fiber to enhance gut health [198] Acting as prebiotics, GPs foster the growth of beneficial gut bacteria, such as *Lactobacillus* and *Bifidobacterium*, promoting digestive health and supporting overall immune function [199]. In recent years, GPs have also gained traction in the beauty and health food markets due to their potent antioxidant and anti-tumor properties, finding applications in anti-aging and cancer-preventive food products [200]. Collectively, the multifunctional properties of GPs position them as a versatile and valuable ingredient in the functional food market, meeting the increasing consumer demand for immune support, gut health, and disease prevention.

Despite the widespread commercial application of GPs in functional foods, several technical and production challenges hinder their optimal utilization in health products. To address these challenges, several approaches can be adopted. First, the low extraction yield of water-soluble GPs—typically ranging between 1 and 3%—remains a major concern. To improve extraction efficiency and purity, and thus reduce production costs, biotechnological fermentation has emerged as a promising solution, as highlighted in numerous studies. Second, further research is needed to explore the bioactivity of GPs under different processing conditions, particularly focusing on their stability during food production and storage. This can be achieved through the development of microencapsulation techniques or the incorporation of antioxidants to protect GPs from environmental degradation. Moreover, improving consumer acceptance remains a key priority. The intrinsic bitterness of GPs poses a challenge in food formulations, necessitating the development of flavor-masking techniques or the combination of GPs with other ingredients to enhance taste profiles. Additionally, the market for GPs health products is highly diverse, yet the lack of activity-based quality standards results in significant variability in product quality and efficacy. Therefore, implementing activity-oriented quality control measures, such as the quantification of specific bioactive components like β-glucans, is crucial to ensuring product consistency and therapeutic effectiveness across the global market.

## Conclusion and future perspectives

GPs have emerged as bioactive compounds with significant pharmacological potential, owing to their complex structural features, such as β-(1 → 3)-D-glucan backbones, β-(1 → 6)-D-glucan branching, and triple-helix conformations. These structural variations, along with diverse monosaccharide compositions, influence their immunomodulatory, anti-tumor, and gut microbiota-regulating properties. Specifically, β-(1 → 6) branching and high molecular weight have been linked to enhanced immune responses by activating PRRs such as Dectin-1 and TLRs, promoting cytokine production and immune cell activation. Additionally, structural elements like triple-helix conformations and functional groups, including acetyl and carboxyl groups, may influence bioactivity, contributing to the observed pharmacological effects.

While previous reviews have advanced the understanding of GPs, they often focus on isolated aspects, such as specific structural features or generalized biological activities, without comprehensively examining the interconnections between structural characteristics, biosynthetic pathways, and functional applications. This review addresses that gap by providing a holistic synthesis of GP structural complexity, SARs, biosynthesis mechanisms, and their applications in medicinal and functional food development. Such an integrative approach offers a clearer understanding of how structural features, such as glycosidic linkage patterns and branching ratios, determine biological activity, while emphasizing the potential of genetic engineering and advanced extraction techniques for optimizing GP yield and functionality.

However, significant challenges remain unresolved. The structural heterogeneity of GPs and variability in extraction methods often lead to inconsistencies in bioactivity reports. Traditional techniques, such as hot water and alkaline extraction, risk degrading polysaccharide structures, compromising biological efficacy. Furthermore, the limited control over biosynthetic pathways, particularly glycosyltransferase activity and branching patterns, hinders large-scale production of GPs with consistent structural features and bioactivity. Addressing these challenges requires innovations in extraction, structural characterization, and biosynthetic optimization to fully realize the therapeutic potential of GPs.

Future research should prioritize several strategies. The adoption of green extraction technologies, including mechanochemical-assisted extraction and deep eutectic solvents, can enhance GP yield while maintaining structural integrity. Genetic engineering approaches, such as overexpressing glycosyltransferase enzymes, hold promise for improving biosynthesis and structural uniformity. Exploring the synergistic effects of GPs with probiotics could uncover novel mechanisms for gut microbiota modulation, enhancing immune health and cancer prevention. Finally, standardizing analytical methods, such as molecular weight profiling and glycosidic linkage analysis, is crucial for ensuring reproducibility and consistency in both research and commercial applications.

By addressing these gaps and building on the comprehensive framework presented in this review, *Ganoderma* polysaccharides hold immense promise for developing functional foods, pharmaceuticals, and personalized health management strategies. This integrative approach not only deepens the scientific understanding of GPs but also bridges traditional herbal medicine with evidence-based modern health sciences.

## Data Availability

No data was used for the research described in the article.
